# From Rapid Place Learning to Behavioral Performance: A Key Role for the Intermediate Hippocampus

**DOI:** 10.1371/journal.pbio.1000089

**Published:** 2009-04-21

**Authors:** Tobias Bast, Iain A Wilson, Menno P Witter, Richard G. M Morris

**Affiliations:** 1 Centre for Cognitive and Neural Systems (CCNS), School of Biomedical Sciences, University of Edinburgh, Edinburgh, United Kingdom; 2 School of Psychology and Institute of Neuroscience, University of Nottingham, Nottingham, United Kingdom; 3 Centre for the Biology of Memory and Kavli Institute for Systems Neuroscience, Norwegian University of Science and Technology (NTNU), Trondheim, Norway; 4 Department of Anatomy and Neuroscience, VU University medical center, Amsterdam, the Netherlands; University of California, Irvine, United States of America

## Abstract

Rapid place encoding by hippocampal neurons, as reflected by place-related firing, has been intensely studied, whereas the substrates that translate hippocampal place codes into behavior have received little attention. A key point relevant to this translation is that hippocampal organization is characterized by functional–anatomical gradients along the septotemporal axis: Whereas the ability of hippocampal neurons to encode accurate place information declines from the septal to temporal end, hippocampal connectivity to prefrontal and subcortical sites that might relate such place information to behavioral-control processes shows an opposite gradient. We examined in rats the impact of selective lesions to relevant parts of the hippocampus on behavioral tests requiring place learning (watermaze procedures) and on in vivo electrophysiological models of hippocampal encoding (long-term potentiation [LTP], place cells). We found that the intermediate hippocampus is necessary and largely sufficient for behavioral performance based on rapid place learning. In contrast, a residual septal pole of the hippocampus, although displaying intact electrophysiological indices of rapid information encoding (LTP, precise place-related firing, and rapid remapping), failed to sustain watermaze performance based on rapid place learning. These data highlight the important distinction between hippocampal encoding and the behavioral performance based on such encoding, and suggest that the intermediate hippocampus, where substrates of rapid accurate place encoding converge with links to behavioral control, is critical to translate rapid (one-trial) place learning into navigational performance.

## Author Summary

The ability to remember locations in space is dependent on an area of the brain called the hippocampus. A much-studied property of neurons in the hippocampus is that they rapidly come to represent or code for specific places—i.e., the hippocampus “learns” places—as animals or humans move through an environment. Here, we identified in rats the hippocampal substrate enabling the translation of place learning into appropriate search and approach behavior (similar to the task of returning to a novel place where you parked your car). We examined the impact of selective lesions to distinct parts of the hippocampus on behavior requiring rapid place learning and on in vivo electrophysiological models of hippocampal learning such as place-related neuronal activity. We showed that translation of rapid place learning into efficient search behavior requires the “intermediate” region of the hippocampus, a region that likely combines anatomical links to visuospatial information processed by the neocortex with links to behavioral control through prefrontal cortex and subcortical sites. In contrast, the so-called “septal” region of the hippocampus, which features the relevant anatomical links to visuospatial information processing, can sustain rapid place learning (as reflected by formation of place-related neuronal firing), but not translate such learning into appropriate search and approach behavior.

## Abstract 3

The translation of hippocampal rapid place learning into successful search behavior requires the intermediate region of the hippocampus, which integrates accurate visuo-spatial processing with behavioral control.

## Introduction

A classical distinction in animal learning theory is that between “learning” and “performance” [1,2]. The processes involved in encoding and storing new information are conceptually distinct from those involved in translating that information into useful behavior. The recent preoccupation with plasticity and encoding mechanisms has sometimes led to this distinction being forgotten, but there are dangers in doing so [3]. The present study focuses on the neuroanatomical substrates of the learning–behavior translation with particular attention to the issue of how rapid place learning results in effective navigational behavior.

**Figure 1:**
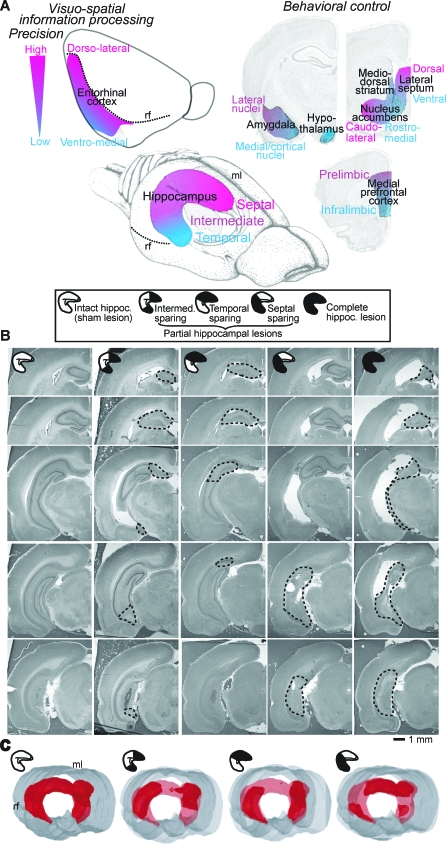
Functional Connectivity of the Hippocampus along the Septotemporal Axis and Ibotenate-Induced Hippocampal Lesions Sparing Different Septotemporal Levels (A) Schematic summary of main functional connectivity of the hippocampus, which is shown in a rat brain with midline (ml) and rhinal fissure (rf) indicated for orientation (center, drawing adapted from Figure 1A of [32]). Hippocampal connectivity is topographically organized along a septotemporal (magenta-blue) gradient. Connections decline either toward the septal pole (close to the septum) or toward the temporal pole (close to the amygdala), so that they are largely restricted to approximately one- to two-thirds starting from either pole. Thus, a differentiation into three partly overlapping domains emerges with distinct sets of connectivity: a septal and temporal region, and, between them, an intermediate region. The septotemporal topography of connectivity to the entorhinal cortex [33,127,128], the main link to visuospatial processing (top left), and to medial prefrontal cortices [127–131] and subcortical sites (mediodorsal striatum and nucleus accumbens [60,132,133], the amygdala [30,118], lateral septum, and hypothalamus [31,134]), which link the hippocampus to behavioral control (i.e., emotional, motivational, sensorimotor, and executive functions) (right), is indicated by magenta-to-blue coloration corresponding to different septotemporal levels of the hippocampus. Note that, within the entorhinal cortex, connectivity to neocortical sites conveying highly processed visuospatial information [135] and the precision at which entorhinal grid cells represent visuospatial information [15,34] decline from dorsolateral to ventromedial parts (magenta to blue). Reciprocal connections with the entorhinal cortex and projections to nucleus accumbens and lateral septum are related to the entire septotemporal hippocampal axis, whereas the projections to the medial prefrontal cortex (prelimbic–infralimbic cortex) and the reciprocal connectivity with the amygdala are restricted to the intermediate and temporal hippocampus. Direct projections to hypothalamic nuclei and mediodorsal striatum largely originate from the temporal portions of the hippocampus (including temporal aspects of the intermediate region). Overall, the septal to intermediate hippocampus, via connections to the dorsolateral portions of the entorhinal cortex, are functionally associated with precise visuospatial processing underlying rapid accurate place encoding; the temporal to intermediate hippocampus, via connections to medial prefrontal cortex and subcortical sites, are functionally linked to behavioral control. A convergence of links to precise visuospatial processing and to behavioral control is essentially restricted to the intermediate hippocampus. (B) Cresyl-violet–stained coronal sections through the hippocampus (hippoc.) of exemplar brains from the five groups in experiments 1 and 2 (from left to right): sham lesion, i.e., an intact hippocampus; partial lesions, sparing continuous chunks of approximately 40% of total hippocampal volume in the intermediate region, at the temporal pole, or at the septal pole; complete hippocampal lesion, i.e., virtually no intact hippocampus (sections are arranged from anterior to posterior, with the most anterior section at the top; lesions were bilateral, but to save space, only one hemisphere is used for illustration). In the hippocampus drawings that are used to indicate the different lesion groups (in this and the following figures), white represents intact tissue, and black indicates lesioned tissue. Lesions occasionally resulted in complete removal of the targeted tissue, mainly in the complete hippocampal lesion group, but more commonly in degenerated tissue without intact neurons (outlined by stippled line). (C) Three-dimensional reconstruction of bilateral hippocampal volume prepared from the coronal sections of the brains with a sham-lesion and with the three different partial hippocampal lesions. Intact hippocampal tissue is shown in dark red. In the brains with partial hippocampal lesions, the volume of the intact (control) hippocampus is indicated in light red for comparison. Apart from the hippocampus, the brain silhouette (gray), with the midline (ml) and the rhinal fissure (rf), is shown for orientation; the silhouette is rendered transparent where it would otherwise cover the view of the hippocampus. The residual hippocampal volumes in the exemplar brains with partial hippocampal lesions shown in (B) and (C) were 41% in the intermediate region, 49% at the temporal pole, and 42% at the septal pole, respectively. In the exemplar brain with intended sparing at the septal pole, there was also some unintended sparing of a small volume (5%) at the temporal pole; this temporal sparing appears relatively large in the depicted view of the three-dimensional reconstruction, but an all-around view of the reconstruction clearly shows that it is only a very small piece of tissue. See Videos S1–S4 for an all-around view of the reconstructions.

Different theories hold that the hippocampus mediates certain forms of rapid learning, including place learning [4–11]. For example, when rats explore a novel environment, hippocampal principal neurons rapidly form place codes, as reflected by place-specific firing [9,12–14]. Recent research has focused on how neocortical visuospatial inputs, entering the hippocampus from the entorhinal cortex [15–18], are processed by different subregions along the transverse and longitudinal axes of the hippocampus to mediate place representations. This work has revealed that different subregions along the hippocampal transverse axis (dentate gyrus, CA3, and CA1) make distinct computational contributions, including rapid encoding and pattern completion (CA3) [19–22], pattern separation (dentate gyrus and CA3) [23,24], and the comparison of new and stored information (CA1) [25–27]. In addition, there is a gradient in the precision of visuospatial encoding along the hippocampal longitudinal, or septotemporal, axis that runs from the septal pole, close to the septum, to the temporal pole, close to the amygdala. Main hippocampal connections display septotemporal topographical gradients; that is, they gradually get weaker toward the septal or temporal pole, so that they are mainly restricted to approximately one- to two-thirds starting from either pole, and thereby an anatomical differentiation emerges into three partly overlapping domains with different sets of connectivity: a septal and temporal region, and, between them, an intermediate region (Figure 1A) [28–32]. With respect to the precision of visuospatial encoding, it is critical that the septal to intermediate hippocampus exhibit strong connectivity to the dorsolateral domain of the entorhinal cortex, which receives strong visuospatial neocortical inputs and where neurons, so-called grid cells, represent visuospatial information at a fine scale (grid-like arranged firing fields of down to 20-cm diameter whose centers are spaced at as little as 30 cm apart); in contrast, the temporal hippocampus is mainly connected to the ventromedial domain of the entorhinal cortex, which receives little visuospatial neocortical input and where fine-grained grid-cell firing patterns are replaced by coarser ones (firing fields of up to 3-m diameter spaced at 5 m) (Figure 1A, top left) [15,18,33,34]. Consistent with this, precise place-field codes for small areas (ca. 10- to 20-cm diameter) in conventionally sized recording environments (ca. 1 m × 1 m or smaller) are restricted to the septal to intermediate hippocampus [25,35–40]. In contrast, most neurons in the temporal hippocampus do not display precise place codes; they show larger and less accurate place fields (several meters in diameter) when recordings are performed in relatively large environments (e.g., an 18-m linear track) [38].

This important work has focused on hippocampal place encoding. However, how are hippocampal place codes translated into behavior? This translation may involve different anatomical routes as a function of whether information has been recently and rapidly acquired or been consolidated in the neocortex through incremental learning and/or over time [10,41,42]. With respect to rapidly and recently acquired place memory, particularly in circumstances when relevant information is changing frequently and cannot be consolidated into neocortex, the translation into behavior is likely to involve hippocampal links to medial prefrontal cortical areas, such as the prelimbic and infralimbic cortex, and subcortical sites, such as the mediodorsal striatum, the nucleus accumbens, the amygdala, lateral septum, and hypothalamus. This is because these brain regions provide access to brain-stem sites mediating motor responses [43–46] and are considered to play key roles in behavioral-control processes (including emotional, motivational, sensorimotor, and executive functions) [30,31,45,47–54]. A key idea is that whereas the septal to intermediate hippocampus mediate fine-grained, accurate place encoding (through interactions with the dorsolateral domain of the entorhinal cortex), strong neuroanatomical and functional links to behavioral control (through connections to prefrontal cortex and subcortical sites) are mainly provided by intermediate to temporal hippocampal regions [29–32,45,55–61] (for neuroanatomical details, see Figure 1A and accompanying legend). It follows that the intermediate hippocampus, where substrates of accurate place encoding converge with direct links to behavioral control, may be critical for the translation of rapid place learning into behavior [47].

Based on these considerations, we hypothesized that the intermediate hippocampus would be both necessary and largely sufficient for behavior on tasks requiring rapid place learning. In contrast, neither the septal nor temporal pole of the hippocampus, each comprising only one of the two complementary sets of functional connectivity (i.e., to dorsolateral domain of entorhinal cortex or to prefrontal cortex and subcortical sites), would sustain such performance. Nevertheless, the septal pole, through its connectivity with the dorsolateral domain of the entorhinal cortex, should be able to mediate the rapid encoding of accurate place representations (even though unable, on its own, to translate this information into action). Importantly, this latter prediction may appear paradoxical from theoretical viewpoints that focus on hippocampal encoding mechanisms alone, but it follows directly from the perspective put forward here emphasizing the distinction between hippocampal encoding and behavioral performance based on such encoding. To test these hypotheses, we examined in rats the impact of highly specific partial hippocampal lesions, sparing distinct parts along the septotemporal axis, on performance of a watermaze task requiring rapid, one-trial, learning of a new place every day and on electrophysiological models of rapid encoding in the septal hippocampus, long-term potentiation (LTP) [62], and place-related cell firing.

An important qualification that influenced our experimental plan is that rapid place learning may have distinct mechanisms from incremental learning over many trials [21–23,41,42,63–67]. Indeed, in contrast to our predictions for a one-trial learning paradigm with a daily changing goal location, previous watermaze studies using reference memory paradigms, in which the same location is learned over many trials across several days, have found that small residuals of hippocampal tissue, especially at the septal pole, can be sufficient to sustain good performance [39,57,68,69]. In fact, slow learning can even occur in the absence of the hippocampus [66,67]. Through incremental training, accurate place information might be acquired [42] and/or consolidated [41] in the neocortex and, from there, be translated into behavior [46]. This route would *not* require direct hippocampal links to behavioral control. Therefore, one of our watermaze experiments contrasted the impact of hippocampal lesions on performance based on rapid, one-trial, learning with that based on incremental learning.

## Results

### Experiment 1: The Intermediate, but Not Septal or Temporal, Hippocampus Can Sustain Performance Based on Rapid Place Learning in the Watermaze

#### Rapid place-learning task and presurgical training.

**Figure 2:**
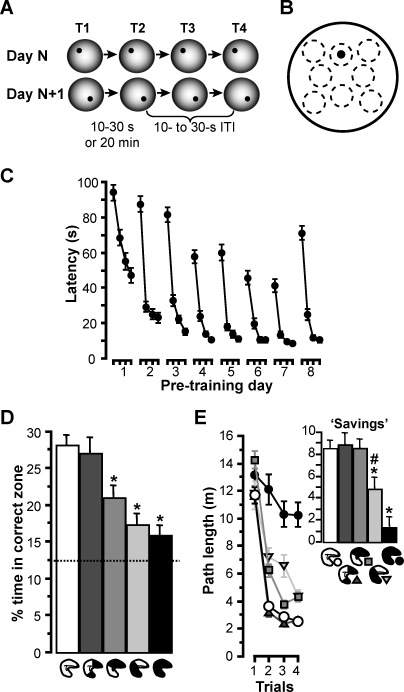
Experiment 1: Rapid-Place Learning Task and Performance after Partial Hippocampal Lesions Sparing the Intermediate, Septal, or Temporal Hippocampus (A) Rats had four daily trials (T1–T4) in the watermaze. The location of the hidden escape platform (black dot) was constant within a day, but the platform was moved to a novel location at the beginning of each day. Thus, rapid place learning during T1 of each day enabled efficient performance during T2 and the subsequent trials. The retention delay between T1 and T2 was 10–30 s or 20 min, whereas all other intertrial intervals (ITI) within a day were 10–30 s. T2 was occasionally run as probe with the platform not coming up until after 60 s, during which time the search preference for the area containing the platform location could be measured. (B) Zone analysis of search preference on probes: eight 40-cm-diameter zones (stippled circles) were defined within the 2-m diameter of the watermaze, including the correct zone, which was concentric with the location of the platform (12-cm diameter; black dot) on T1 of the day. The zones were nonoverlapping, evenly spaced, and symmetrically arranged. The time rats spent in the different zones during the 60-s probe trial was measured, and the percentage of time spent in the correct zone was calculated as: (time in the correct zone/time in all eight zones) × 100%. By chance, i.e., during random swimming, this value should be 12.5%, whereas higher values indicate a search preference for the correct zone based on one-trial place learning. (C) To illustrate the task principle, latencies (mean ± SEM) to reach the platform are plotted for T1–T4 across the eight pretraining days preceding the lesion surgery. Rats (*n* = 89) typically showed high latencies on T1, reflecting searching for the novel platform location, and substantially reduced latencies during subsequent trials, reflecting more efficient navigation based on the use of information learned during the preceding trials (main effect of four daily trials, *F*_3,252_ = 419.8, *p* < 0.0001). Especially striking were the latency reductions (savings) from T1 to T2 (*t*_88_ = 19.05, *p* < 0.0001), demonstrating one-trial learning. Across days, mainly the first 4 d, there was an overall decrease in latencies (main effect of days, *F*_7,588_ = 84.9, *p* < 0.0001) and improved latency reductions between trials (interaction days × trials: *F*_21,1764_ = 5.09, *p* < 0.0001), indicating learning of the general task demands (swimming, moving away from pool wall, win-stay rule). Data were collapsed for the two different T1–T2 delays (10–30s or 20 min), which were used equally often and for half of the rats each on every day. On days 4 and 8, T2 was run as probe. (D and E) Postsurgical performance: (D) percentage time (%time) in correct zone (mean ± SEM) on probes and (E) path lengths (mean ± SEM) to reach the platform on T1–T4, as well as path-length reductions from T1 to T2 (savings, inset graph) in sham-lesioned rats, in rats with approximately 40% of residual hippocampal volume in the intermediate, temporal, or septal hippocampus, and in rats with complete hippocampal lesions (compare Figure 1B and 1C). Group differences: an asterisk (*) indicates different from groups without asterisk (*p* < 0.025); a number sign (#) indicates different from group with complete hippocampal lesions (*p* < 0.025); no additional significant differences (*p* > 0.05). All groups showed higher than chance (indicated by stippled line) search preference and significant path-length reductions from trial 1 to 2 (*t* > 4.4, *p* < 0.04), except for the one with complete hippocampal lesions (*t* < 3.0, *p* > 0.16).

We used a modification of the delayed-matching-to-place watermaze task [70]. On four daily trials, rats had to find a hidden escape platform that was moved to a novel location every day. They could learn the location on trial 1 of a day, and use this place memory for efficient behavior on the remaining trials 2 to 4 of a day. Analysis mainly focused on trial 2, which was run either 10–30 s (minimal time required for the experimenter to complete one trial and start the next) or 20 min after trial 1 to assess the possible delay dependence of lesion effects. Trials 3 and 4 mainly served to reinforce the task's win-stay rule, and were run 10–30 s apart. Rapid, one-trial, place learning on this task is indicated by savings, i.e., a reduction in escape latency between trial 1 and 2 of each day [70]. However, latencies strongly depend on chance, and may furthermore be reduced substantially through systematic search strategies not relying on place memory, by the use of single beacon cues, and by coarse estimates of position and/or sense of direction mediated partly by extrahippocampal structures [71]. Therefore, as an important modification of the original task [70], we occasionally ran trial 2 as probe with the platform not becoming available until after 60 s. During this 60-s period, the rats' search preference for the correct area, a reliable and sensitive index of allocentric place memory, was analyzed using the zone method and expressed as percentage of time in the correct zone (Figure 2A and 2B).

Before surgery, rats were trained on this rapid place-learning task for 8 d (analysis of presurgical performance was restricted to the 89 rats that were included in the final analysis based on the lesion examination at the end of the experiments). They came to express one-trial place memory reliably, as indicated by the robust savings between trial 1 and 2 (Figure 2C) and—when trial 2 was run as probe on days 4 and 8—by a percentage of time spent in the correct zone that was more than twice as high (mean ± standard error of the mean [SEM]: 28.5 ± 1.0%) as expected based on chance (12.5%; *t*_88_ = 17.78, *p* < 0.001). At this stage, performance was independent of the retention delay (10–30 s or 20 min) between trial 1 and 2 (Figure S1). The rats were then divided into performance-matched groups to receive the different surgical treatments (Figure S1).

#### Partial hippocampal lesions selectively sparing intermediate, temporal, or septal hippocampus.

Rats received partial or complete cytotoxic hippocampal lesions (induced by stereotaxic ibotenic-acid injections) or sham surgery (Figure 1B and 1C). Three groups received partial hippocampal lesions sparing approximately 40% of total hippocampal volume (dentate gyrus, CA1–3; 100% was defined as the mean of the sham-operated control group) at the septal pole (mean ± SEM: 43.3 ± 1.0%; range: 36.4% to 48.4%; *n* = 13), at the temporal pole (41.6 ± 1.6%; 27.1% to 56.1%; *n* = 22) pole, or in the intermediate region (40.1 ± 2.0%; 27.6% to 54.1%; *n* = 14). Particular attention was paid to ensuring that the volume of spared tissue was equivalent between the partial hippocampal groups in order to avoid behavioral differences due to volume effects. In addition, a group with complete hippocampal lesions (residual hippocampal volume: 1.9 ± 0.4%; 0% to 5.4%; *n* = 14), and a sham-operated group, with intact hippocampus (100.0 ± 1.4%; 83.7% to 112.4%; *n* = 26), were included (*n* refers to the number of rats that had acceptable lesions and were included in data analysis; for additional lesion analysis, see Text S1, Supplementary Results 1). Three-dimensional reconstruction of the hippocampal sparing in exemplar brains of the groups with partial hippocampal lesions revealed bilateral, relatively symmetric residual tissue chunks of similar size, at different locations along the septotemporal axis (Figure 1C, Videos S1–S4).

#### Postlesion performance on the rapid place-learning task.

Of the lesioned groups, only the one with an intact intermediate hippocampus was unimpaired on the rapid place-learning task (Figure 2D and 2E). There was an overall dependence of performance on the retention delay between trials 1 and 2 (Figure S2), but group differences were all delay independent (interactions involving groups × delays: *F* < 1.7, *p* > 0.17); therefore, the main analysis focused on data averaged over both delays. Rats with a residual intermediate hippocampus showed similarly strong preference for the correct zone as the sham group during trial 2 (run as probe) of postsurgical days 4 and 8; in contrast, rats with only the septal or temporal pole of the hippocampus were strongly impaired, and rats with complete hippocampal lesions showed no preference at all (main effect of group: *F*_4,84_ = 9.0, *p* < 0.0001; post hoc comparisons and comparisons to chance: see figure) (Figure 2D). Path-length savings between trial 1 and 2 (which we analyzed instead of latencies, as some of the lesions affected swim speed; see below) were impaired in rats with only the septal pole spared, and abolished by complete hippocampal lesions (interaction groups × trials: *F*_12,252_ = 8.3, *p* < 0.0001; main effect of group on “savings”: *F*_4,84_ = 10.7, *p* < 0.0001; post hoc comparisons and comparisons to chance: see figure) (Figure 2E). Here, and in all other experiments of the present study, performance measures did not correlate with the residual hippocampal volume in any of the different groups (all *r* < 0.41, *p* > 0.15), which was not unexpected, as we endeavored to keep variability in lesion size to a minimum. The results support the hypothesis that only the intermediate hippocampus, where connectivity to the dorsolateral domain of the entorhinal cortex converges with direct links to prefrontal cortex and subcortical sites, can sustain normal performance based on rapid place learning, whereas the septal or temporal hippocampus, each comprising only one of the two complementary sets of connections, cannot.

### Experiment 2: Incremental versus Rapid Place Learning

**Figure 3:**
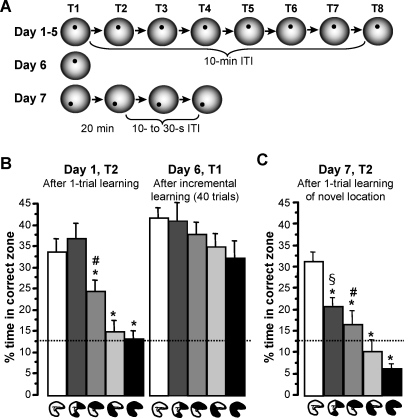
Experiment 2: Performance Based on Incremental, as Compared to Rapid, Place Learning after Partial Hippocampal Lesions Sparing the Intermediate, Septal, or Temporal Hippocampus (A) Rats were trained to one constant platform location on eight daily trials (T1–T8) for 5 d, with T2 and T6 of each day run as probes to closely monitor performance improvements. The intertrial interval (ITI) was 10 min. On day 6, one additional probe trial was run to assess performance based on incremental place learning across the five preceding days unconfounded by within-day learning. On day 7, rats were retested on the rapid place-learning task, with four trials to a novel location and T2 run as probe. The retention delay between T1 and T2 was 20 min, and the other ITIs were 10–30 s. (B) Search preference for the correct zone (mean ± SEM) after one-trial place-learning (day 1, T2) and after 40 trials of slow, incremental learning (day 6, T1) in the different lesion groups (compare Figure 1B and 1C). ANOVA indicated significant group differences only on day 1, T2 (see main text), and results of the post hoc comparisons are indicated: an asterisk (*) indicates different from groups without an asterisk (*p* < 0.015); a number sign (#) indicates different from the group with the hippocampal residual at the septal pole and the one with complete hippocampal lesions (*p* < 0.04); no additional significant differences (*p* > 0.4). On day 1, T2, search preference for the correct zone was above chance (stippled line) in rats with sham-lesions and with hippocampal residuals in the intermediate region or at the temporal pole (*t* > 3.1, *p* < 0.01), but not in rats with hippocampal residuals at the septal pole or with complete hippocampal lesions (*t* < 1.5, *p* > 0.19). In contrast, all groups showed a significant preference for the correct zone on day 6, T1 (*t* > 4.8, *p* < 0.0005). (C) When retested for performance based on one-trial place learning on day 7, T2, there were marked group differences in search preference for the correct zone (mean ± SEM): an asterisk (*) indicates different from sham-operated group (*p* < 0.025); a number sign (#) indicates different from group with complete hippocampal lesions (*p* < 0.025); a section symbol (§) indicates different from group with complete hippocampal lesions and group with only the septal pole spared (*p* < 0.01); no additional significant differences (*p* > 0.09). Sham-operated rats and those with a residual intermediate hippocampus showed a significant preference for the correct zone (*t* > 3.9, *p* < 0.002), whereas rats with hippocampal residuals at septal or temporal pole did not differ from chance (*t* < 1.2, *p* > 0.26), and rats with complete hippocampal lesions spent even less time in the correct zone than expected by chance (*t*_13_ = 4.93, *p* < 0.0004).

At first glance, our results may appear inconsistent with previous findings that rats with only 20%–40% of residual hippocampal volume at the septal or temporal pole can express similar place memory in the watermaze as control groups [68,69]. However, in these studies, incremental learning was possible, as the rats were trained to the same place over many trials. To compare performance based on rapid versus incremental learning, we therefore trained the different lesion groups with one constant platform location for 5 d, with eight trials per day, and trials 2 and 6 of each day run as probes to closely monitor performance. On day 6, one additional probe trial was run to assess incrementally acquired long-term memory independent of within-day learning. On day 7, a subset of the rats (the last four of five replications, a total of 72 rats: 20 sham-operated, 13 with residual intermediate hippocampus, 15 with residual temporal pole, 10 with residual septal pole, and 14 with complete hippocampal lesions) was retested for performance based on rapid place learning, with a novel platform location and four trials (trial 2 run as probe); this retest was included to rule out that nonspecific recovery due to extended postsurgical training might account for any better performance after incremental place learning (Figure 3A).

The differential effects of hippocampal damage on performance based on rapid versus incremental place learning were strikingly revealed by search preference on trial 2 of the first day, i.e., after one learning trial, and on trial 1 of day 6, i.e., after 40 trials run across the preceding 5 d (Figure 3B). After only one learning trial, rats with sham lesions or residual intermediate hippocampus focused their search on the correct zone, whereas rats with only the septal or temporal pole, or with complete hippocampal lesions were impaired (Figure 3B, left), replicating the results from experiment 1 (compare Figure 2D); in contrast, after slow, incremental learning over 40 trials, groups did not differ anymore (Figure 3B, right) (interaction group × probe trial [day 1, trial 2, vs. day 6, trial 1]: *F*_4,84_ = 2.5, *p* < 0.05; main effect of group, day 1, trial 2: *F*_4,84_ = 11.0, *p* < 0.0001, post hoc comparisons: see figure; main effect of group, day 6, trial 1: *F*_4,84_ = 1.4, *p* > 0.22). Interestingly, the sham-operated rats and all groups with partial hippocampal lesions showed some rapid within-day performance improvements that were not carried over to the next day, so that latencies on trial 1 of a day tended to be higher than on the last trial of the preceding day and percentage of time in the correct zone tended to be lower on the first probe of a day (trial 2) than on the last probe (trial 6) of the previous day. In contrast, rats with complete hippocampal lesions gradually improved across trials and days (Figure S3). In previous studies, using incremental training in the watermaze, rats with residual 20%–40% of hippocampal volume at the septal pole did not differ from sham-lesioned rats at a stage when performance was still hippocampus dependent, i.e., impaired in rats with complete hippocampal lesions [69] (under certain training conditions, this also pertained to rats with 20%–40% of hippocampal volume restricted to the temporal pole [68]). These findings were confirmed in the present experiment: on trial 1 of day 4, paths were longer in rats with complete hippocampal lesions (*F*_4,84_ = 2.76, *p* < 0.03), as compared to sham-operated rats (*p* < 0.01) and the partial-lesion groups (*p* < 0.03), excepting rats with remnants at the temporal pole for which the difference just failed to reach significance (*p* = 0.054); the partial lesion groups did not differ from each other or the sham-lesioned rats (*p* > 0.3) (Figure S3A). These results demonstrate that hippocampal contributions are especially required for behavior requiring rapid place learning; if incremental place learning is possible, good navigational performance can be achieved with hippocampal residuals at the septal or temporal pole, and eventually even without a hippocampus.

In stark contrast to the similarly good performance across groups after incremental place learning (Figure 3B, right), there were again marked group differences when rats were subsequently retested for performance based on rapid place learning on day 7 (Figure 3C; for path-length data, see Figure S4). During the probe on trial 2, after one training trial to a novel location, only the sham-operated rats and those with a residual intermediate hippocampus significantly preferred the correct zone; rats with only the septal or temporal pole of the hippocampus did not differ from chance, and rats with complete hippocampal lesions spent even less time in the correct zone than expected by chance (as they kept searching for the platform in the previous, incrementally learnt location, but had not learnt the novel location) (main effect of groups: *F*_4,67_ = 18.52, *p* < 0.0001; between-groups post hoc comparisons and comparisons to chance: see Figure 3C). These results corroborate the special importance of the hippocampus for performance based on rapid place learning. Interestingly, even though the group with an intact intermediate hippocampus was still the best of all groups that had any damage to the hippocampus, it was significantly impaired as compared to the sham-operated group (*p* < 0.0025), possibly related to interference from the preceding incremental place-learning task.

### Experiments 3 and 4: Intact Entorhinal Cortex–Dentate Gyrus Plasticity and Intact Place-Related CA1 Cell Firing in Hippocampal Residuals at the Septal Pole

Rats with hippocampal residuals at the septal pole showed poor performance on the rapid place-learning task, whereas rats with a residual intermediate hippocampus displayed largely intact performance. According to our hypothesis, this finding does not reflect that a residual septal pole is less capable of accurate place encoding/learning than a residual intermediate hippocampus (but rather that a residual septal pole, lacking the links of the intermediate hippocampus to behavioral-control sites, is incapable of translating place learning into performance). Indeed, in the intact hippocampus, neurons at the septal pole encode visuospatial information at even higher precision than neurons in the intermediate region [37,38,40]. We predicted that such encoding in the septal hippocampus would be unaffected by neurotoxic lesions to the rest of the hippocampus, given that such lesions should not affect the relevant entorhinal–hippocampal interactions [16,18,39,71]. To test this point, we examined properties of the residual hippocampal circuitry at the septal pole using electrophysiological models of information encoding, predicting that synaptic plasticity and place-field encoding would be essentially normal.

**Figure 4:**
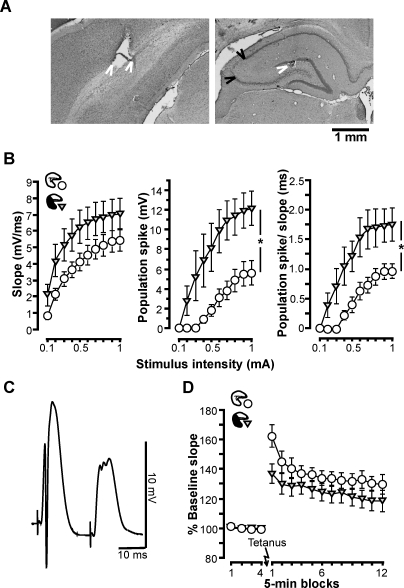
Experiment 3: Functional and Plastic Entorhinal Cortex–Dentate Gyrus Connectivity in Hippocampal Residuals at the Septal Pole (A) Electrolytic lesions (white arrowheads) marking the stimulation site in the perforant path (bipolar electrode, left) and the recording site in the ipsilateral dentate gyrus in cresyl-violet–stained coronal sections. Photos are from a brain with partial hippocampal lesion sparing the septal pole (35% of hippocampal volume), and damage to CA3 (black arrowheads) is visible at the level of the recording site. (B) Input–output curves recorded from rats with sham lesions or with partial hippocampal lesions sparing residuals at the septal pole: different measures of the evoked field response (mean ± SEM) are plotted as function of the stimulus intensity (0.1–1 mA). An asterisk (*) indicates significant main effect of group (*p* < 0.03). (C) Paired-pulse inhibition in a septal hippocampal residual: field responses evoked by paired stimulation (0.5 mA, 20 ms apart; average of 15 samples). Note the large population spike in the first response and its virtual absence in the second response. The residual hippocampal volume in this case was 27%. (D) Brief tetanic stimulation induces similar LTP of the evoked response in rats with sham lesions and with partial hippocampal lesions sparing residuals at the septal pole. The evoked response is expressed as percentage baseline slope (mean ± SEM).

In experiment 3, evoked field potentials in the perforant path–dentate gyrus pathway, a main entorhinal input to the hippocampus, were recorded from the septal hippocampus of anesthetized rats that were sham-lesioned, i.e., had an intact hippocampus (mean ± SEM: 100 ± 3.5%, range: 84.4%–107.5%; *n* = 6), or had received partial hippocampal lesions sparing only the septal pole (34.1 ± 1.7% of control hippocampus, 27.1%–41.7%; *n* = 7) (Figure 4A). Input–output curves indicated no significant difference in field-potential slopes between these groups (*F*_1,11_ = 2.72, *p* > 0.12) (Figure 4B, left). However, in lesioned rats, perforant-path stimulation triggered neuronal firing more readily than in sham-operated rats, as indicated by significantly higher population spike amplitude and population spike/slope ratio (main effects of group: *F*_1,11_ > 7.16, *p* < 0.02) across all stimulation intensities, except for the lowest (interactions group × intensity: *F*_9,99_ > 2.02, *p* < 0.05) (Figure 4B, middle and right). These findings may reflect that lesion of the temporal and intermediate hippocampus removed feed-forward inhibition by longitudinally projecting inhibitory interneurons, which have been suggested to support coordination of neuronal firing along the septotemporal axis in the intact hippocampus [72–74]. Feedback inhibition, as indicated by paired-pulse inhibition [75], could readily be demonstrated in hippocampal residuals at the septal pole (Figure 4C), ruling out a general hyperexcitability. Importantly, tetanization resulted in robust LTP in both groups (Figure 4D). Slopes measured 55–60 min after the tetanus were significantly increased as compared to the 5 min preceding the tetanus in both groups (*t* > 2.87, *p* < 0.05) and did not differ between groups (*t*_11_ = 1.15, *p* = 0.27). Thus, hippocampal residuals at the septal pole displayed normal synaptic plasticity.

**Figure 5:**
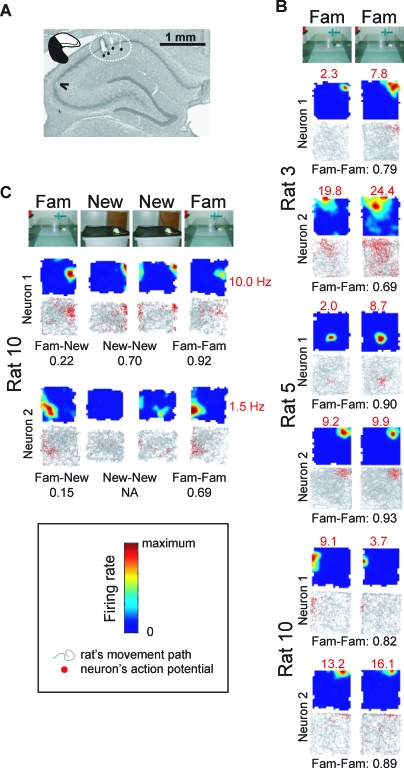
Experiment 4: Place-Related Firing of Neurons in Hippocampal Residuals at the Septal Pole (A) Cresyl-violet–stained coronal section showing the tracks of the four tetrode bundles (circled by stippled line; individual tracks indicated by black dots) used to record place-related activity from the CA1 region of the septal hippocampus; tracks were marked by passing an electric current through the tetrodes after completion of the recordings. The section is from rat 10, which had a hippocampal residual of 26% at the septal pole. A slight disruption of the CA3 layer is visible (black arrowhead), indicating the beginning of the lesion to intermediate and temporal hippocampus. (B) CA1 cells in hippocampal residuals at the septal pole show accurate and stable place-related firing in a familiar environment (Fam): Firing fields of complex-spike cells recorded from three rats (3, 5, and 10) with hippocampal residuals at the septal pole during two successive trials in the familiar environment. The color-coded firing-rate maps and, below, the location of spikes (red) on the rat's trajectory (gray) are shown for the two cells of each rat with the highest spatial-information score. The peak firing rate for each trial is indicated in red numbers above and the correlation between the firing-rate maps for the two successive trials is shown in black below. Note that neurons often changed their peak firing rate between trials, but that the firing positions remained stable. See Figure S5A and S5B for additional cells recorded from the rats with hippocampal residuals at the septal pole and for cells recorded from a control group with intact hippocampus. (C) CA1 cells in hippocampal residuals at the septal pole rapidly change their firing pattern in a new environment. Firing fields of two complex-spike cells recorded from one rat with hippocampal residuals at the septal pole (rat 10; the two neurons are not identical with the two neurons whose firing patterns are shown in [B]) during a succession of trials in the familiar (Fam) and a new (New) environment. All firing-rate maps are scaled to the same maximum firing rate, equal to the maximum of all four trials and indicated in red to the right; correlations between the firing-rate maps for different trials are indicated in black below. Note that neuron 1 forms a new place field in the new environment, whereas neuron 2 becomes nearly silent. See Figure S6 for additional cells from rat 10 and from two control rats with intact hippocampus.

In experiment 4, single-unit recordings revealed accurate and stable place-related firing in hippocampal residuals at the septal pole (Figure 5A) in rats that were foraging in large open fields surrounded by prominent distal visual cues. Starting with a highly familiar environment, 42 putative pyramidal neurons were recorded from three rats with partial hippocampal lesions sparing only the septal pole (mean ± SEM: 30.4 ± 2.3% of control hippocampus, range: 25.8%–33.02%). These neurons exhibited highly place-related firing that was stable between two recording trials in the familiar environment (typically separated by 30 to 60 min) (Figures 5B and S5A); spatial firing was similar to that of pyramidal neurons (*n* = 28) recorded from the septal pole of an intact hippocampus (100 ± 11.5%, 79.9%–119.8%) in control rats (*n* = 4) (Figure S5B).

Quantitative measures of place-related firing (average and peak rates, spatial information, and sparsity) and its stability between two successive trials in the familiar environment (correlation, changes in average and peak firing rate, movement of firing peak) were calculated (mean ± SEM) and compared between groups (data of different cells recorded from the same rat were averaged, so that each rat contributed one value to the group means) (Table S1). Only the stability measures differed, with a lower between-trials correlation of firing patterns in cells from septal remnants (0.75 ± 0.03, as compared to 0.88 ± 0.02 in the intact hippocampus; *t*_5_ = 3.58, *p* < 0.02; correlation coefficients were subjected to Fisher's *z*′-transformation before the *t*-test). This difference could be accounted for by a relatively high between-trials change in peak firing rate in place cells of septal remnants (0.28 ± 0.04; compare also Figures 5A and S5A), which was twice as high as in cells from an intact hippocampus (0.14 ± 0.03; *t*_5_ = 3.17, *p* < 0.025). Importantly, the position of peak firing was equally stable in both groups (movement of firing peak between trials in centimeters: septal remnants, 14.3 ± 3.6; intact hippocampus, 16.5 ± 4.2, *t*_5_ < 1).

Finally, when exposed to a new environment, CA1 pyramidal neurons (*n* = 9) in a hippocampal residual at the septal pole rapidly changed their firing patterns reflecting normal remapping [13] (Figures 5C and S6). Thus, overall, neurons in hippocampal residuals at the septal pole displayed normal accurate and rapid place encoding.

### Experiment 5: The Intermediate Hippocampus Is Necessary for Performance on the Rapid Place- Learning Task

Experiment 5 addressed whether the convergence of substrates for rapid place encoding with those for behavioral control in the intermediate hippocampus was really critical for performance based on rapid place learning, as implied by our conceptual framework. Alternatively, independent, parallel contributions of substrates mediating rapid place encoding and of links to behavioral control at the septal and temporal tip of the hippocampus, respectively, could mediate performance. To decide between these two alternatives, our last experiment tested whether residuals at the septal and temporal tip (ca. 20% of total hippocampal volume spared at each tip), separated by a lesion to the intermediate hippocampus, could sustain performance. Thus, experiment 5 is the “mirror image” of experiment 1: whereas experiment 1 established that hippocampal tissue restricted to the intermediate hippocampus was sufficient for performance based on rapid place learning, experiment 5 tested whether damage restricted to this area causes an impairment in performance. Importantly, whereas different septotemporal levels are connected through intrahippocampal longitudinal projections, there is no evidence for direct links between the septal and temporal 20% of the hippocampal volume, and the only intrahippocampal connection between these septal and temporal tips may be by way of the intermediate region, which receives afferents from the septal tip and projects to the temporal tip [28,76–80]. Thus, the septal and temporal tips spared by the lesions made to the intermediate hippocampus in the present experiment would essentially be disconnected.

**Figure 6:**
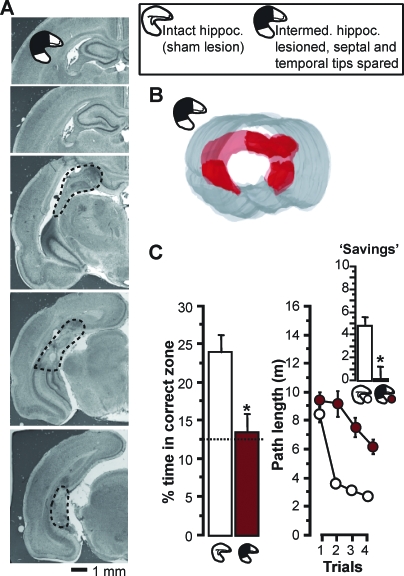
Experiment 5: Lesions to the Intermediate Hippocampus, Sparing the Temporal and Septal Tip, Abolish Performance Based on Rapid Place Learning (A) Cresyl-violet–stained coronal sections through the hippocampus of one exemplar brain with lesion to the intermediate hippocampus (arranged from most anterior, top left, to most posterior, bottom right; degenerated tissue without intact neurons outlined by stippled line). (B) Three-dimensional reconstruction of bilateral hippocampal volume in the same brain (compare Figure 1C for explanation and abbreviations). The residual hippocampal volume in the exemplar brain was 43%: 21% at the temporal and 22% at the septal tip. See Video S5 for an all-around view. (C) Performance on the rapid place-learning task in the watermaze (compare Figure 2A), as reflected by percentage time (% time) in correct zone on probes and path lengths on T1–T4, as well as path-length reductions from T1 to T2 (savings, inset graph) (all data: mean ± SEM), in rats with lesions to the intermediate hippocampus and in sham-operated rats. An asterisk (*) indicates significant group differences (*p* < 0.005). Sham-operated rats showed higher than chance search preference and a significant path-length reduction from trial 1 to 2 (*t*_11_ > 5.23, *p* < 0.0003), but rats with lesions to the intermediate hippocampus did not (*t*_10_ < 0.41).

Rats were pretrained on the rapid place-learning task as described for experiment 1 and then divided into two performance-matched groups (Figure S7). One group received sham surgery (hippocampal volume, mean ± SEM: 100.0 ± 2.1%, range: 90.7%–115.0%; *n* = 12); the other one had lesions to the intermediate hippocampus, sparing approximately 20% of hippocampal volume each at the septal (19.9 ± 1.2%, 12.3%–25.4%) and the temporal (22.1 ± 0.75%, 8.3%–27.1%) tip of the hippocampus (*n* = 11) (Figure 6A and 6B; Video S5) (*n* refers to the rats included in data analysis; for additional lesion analysis, see Text S1, Supplementary Results 1). Lesions to the intermediate hippocampus virtually abolished performance (Figure 6C). Performance did not significantly depend on the retention delay between trial 1 and 2 (Figure S8), and group differences were delay-independent (interactions involving groups × delays: *F* < 1), so the analysis focused on data averaged over both delays. In contrast to sham-operated rats, rats without the intermediate hippocampus showed no search preference for the correct zone on probe trials (main effect of group: *F*_1,21_ = 10.9, *p* < 0.004) and no savings between trial 1 and 2 (interaction groups × trials: *F*_3,63_ = 6.8, *p* < 0.001; main effect of group on savings: *F*_1,21_ = 14.3, *p* < 0.002; comparisons to chance: see figure) (Figure 6C). Thus, separated hippocampal residuals at the temporal and septal tip cannot sustain performance.

### Increased Swim Speed after Lesions Involving Damage to the Temporal Half of the Hippocampus

In each of the watermaze experiments (experiments 1, 2, and 5), lesions affecting the temporal half of the hippocampus increased swim speed (Text S1, Supplementary Results 2). This increase is in line with previous observations [56] and corroborates the close association of the temporal half of the hippocampus with sites mediating motor function [55].

## Discussion

The present findings show that the intermediate hippocampus is critical for watermaze performance based on rapid, one-trial, place learning with the goal location changing each day. Partial hippocampal lesions sparing the intermediate hippocampus (ca. 40% spared of total volume) left performance on the one-trial task largely intact, whereas partial hippocampal lesions sparing the septal or temporal pole (ca. 40% of total volume) impaired performance. Performance was abolished by lesions to the intermediate hippocampus, sparing the septal and temporal tips (ca. 20% of hippocampus each). Although a residual hippocampal circuitry at the septal pole could not sustain behavioral performance based on rapid-place learning, evoked-potential and place-cell recordings revealed it had functional and plastic connectivity to the entorhinal cortex and could rapidly and accurately encode visuospatial information. Thus, residual circuitry at the septal pole can “learn” rapidly, but cannot alone translate a rapidly acquired place representation into appropriate behavior. Such translation, our data suggest, requires the intermediate hippocampus, where substrates of rapid place learning converge with links to behavioral-control functions.

### Rapid Learning of Changing Locations versus Incremental Learning of a Stable Location

Hippocampal lesions caused a quite different pattern of results on the rapid, one-trial, place-learning task, in which the goal location changed every day, than on the incremental-learning task, in which rats were trained to the same location over many trials across days and which belongs to the more commonly used reference-memory paradigms. A conceptually important difference between these two watermaze tasks relates to the relevance of hippocampal information encoding and neocortical memory acquisition/consolidation.

On the rapid-learning task, the goal location is constantly changing from day to day, requiring the rapid encoding of stimuli and their relations, for which the hippocampus and its synaptic plasticity are critical [8,9,21–23,41,42,64,66,70,81,82]. Information about the goal location must be constantly updated by hippocampal encoding mechanisms and, from the hippocampus, secure direct access to behavioral control systems. Consistent with this, we found that when rapid, one-trial, learning of a novel place is required, the hippocampus, especially the intermediate part, is essential for effective performance (independent of retention delay, see Text S1, Supplementary Discussion 1).

On the incremental-learning task, in contrast, all relevant information is stable over time. It has been suggested that under these circumstances, information can be consolidated from the hippocampus into the neocortex [41,83,84] or gradually be acquired by the neocortex [42]. From neocortical storage sites, effective behavioral control is possible via connections bypassing hippocampal pathways [46]. Although hippocampal circuitry may normally contribute to incremental place-learning tasks, and even small residuals of such circuitry, especially at the septal pole, may speed up acquisition (discussed below), our experiment 2 demonstrated that even rats with complete hippocampal lesions could eventually, albeit at a much slower rate than a control group, come to display accurate place navigation (as shown on probe trials by their focused search preference in a small 40-cm-diameter zone centered on the platform location). This confirms and extends previous demonstrations of relatively intact performance in the watermaze after extended incremental training in rats with complete hippocampal lesions [66,67] (also see Text S1, Supplementary Discussion 2). Humans with hippocampal lesions, who show marked impairments in place and declarative memory, can also come to express accurate place [85] and semantic [86,87] memory, when incremental learning is possible. Without the hippocampus, initially coarse neocortical representations of relevant information, for example of places in the entorhinal cortex [15,17], may slowly be sharpened into accurate, nonoverlapping representations through incremental learning [42] and then be translated into behavior via direct neocortical connections. Hippocampal circuitry may normally contribute to incremental place-learning tasks; however, it is not absolutely required, and relatively good performance on such tasks can eventually be achieved without the hippocampus, albeit very slowly (also compare [66]).

### Neither the Septal nor the Temporal Pole of the Hippocampus Can Sustain Normal Performance Based on Rapid Place Learning

Rats with hippocampal tissue restricted to the septal or temporal pole were markedly impaired on all tests requiring rapid, especially one-trial, place learning. Both groups exhibited deficits in the main performance measure, search preference during probes, whereas only the rats with tissue restricted to the septal pole showed impaired path-length savings.

A number of previous studies have found that rats with partial hippocampal lesions sparing only the septal pole can display efficient performance on place-learning tasks, whereas rats with residuals at the temporal pole cannot (for review, see [39,57]). For example, after 32 trials (eight trials/day, 4 d) to the same platform location in the watermaze, rats with hippocampal remnants of 20%–40% of total volume at the septal pole showed relatively normal performance, whereas rats with even 40%–60% spared hippocampal volume at the temporal pole were substantially impaired [68,69]. What accounts for this advantage of rats with septal hippocampal sparing on the reference-memory version of the watermaze task? Residual septal hippocampal circuitry is capable of rapid place encoding, as revealed by our place-cell recording experiments, even though it lacks the connectivity to relate place codes directly to behavioral-control functions. However, as all relevant place information on the reference-memory version of the watermaze task is stable, place information acquired within hippocampus may, through incremental learning and the process of systems-level consolidation, become established, or “interleaved,” into neocortical networks [8,41,83,84,88,89], which can then translate such stored information into behavior. A residual septal pole may accelerate performance acquisition on watermaze reference-memory tasks, because neocortical learning aided by rapid septal hippocampal place encoding is faster than purely neocortical learning (even though eventually even rats with complete hippocampal lesions can come to show good performance). Importantly, however, several studies have found that rats with 30%–50% of hippocampal volume at the septal pole show performance deficits at early stages of incremental place learning tasks [90], i.e., when performance improvement likely depends on rapid learning and its translation into action through direct hippocampal links to behavioral control, and that these deficits diminish with additional training [68,91,92]; performance deficits have also been indicated on watermaze tests of one-trial place learning as reduced savings [92,93] (but see [56]). Although the boundary conditions under which the septal or temporal pole of the hippocampus can sustain performance on place-learning tasks remain to be clarified (also see [68]), our new findings demonstrate that neither region alone can sustain normal navigational performance based on rapid, one-trial, place learning.

Our findings reflect, we suggest, that the temporal region of the hippocampus cannot form accurate place codes, as it lacks close links to the dorsolateral domain of the entorhinal cortex, whereas the septal region, due to its connectivity with this part of the entorhinal cortex, can rapidly form accurate place codes, but not translate them into behavioral control, due to the lack of connectivity to prefrontal cortex and subcortical sites (see Introduction and Figure 1A). Several lines of evidence, comprising recordings of single-unit firing and of multisite coordinated activity, as well as studies using lesion and pharmacological manipulations of relevant brain sites (including disconnection approaches), support the notion that hippocampal interactions with the dorsolateral domain of the entorhinal cortex [16,94–96] and with medial prefrontal cortex and subcortical sites, specifically the mediodorsal striatum and nucleus accumbens [47,48,51,97–104], are important for performance on place-learning tasks. In support of our emphasis on the importance of hippocampal–prefrontal/subcortical interactions in interpreting our results, the following findings are noteworthy. First, lesions to medial prefrontal cortex or mediodorsal striatum in rats [50] or glutamate-receptor blockade in the nucleus accumbens of mice [105] impair performance on watermaze rapid place-learning tasks similar to the one used in the present study. Second, using crossed unilateral lesions or pharmacological manipulations of the relevant sites, it was demonstrated that disconnection of the hippocampus from medial prefrontal cortex [48,103,104] or prefrontal dopamine transmission [99], from mediodorsal striatum [101], or from nucleus accumbens [48] impaired rats' performance on different dry-land or watermaze tasks requiring rapid place learning. Third, electrophysiological recording studies showed that neuronal activity in the hippocampus is coordinated with activity in medial prefrontal cortex [97,98], mediodorsal striatum [100], and nucleus accumbens [102] while rats are using rapidly encoded place information for efficient foraging behavior. Other subcortical sites, such as amygdala, lateral septum, and hypothalamus, which have been implicated in behavioral control and display strong connectivity to temporal and intermediate parts of the hippocampus [30,31], may also interact with the hippocampus to translate place learning into behavior, but this possibility remains to be tested. The tendency, observed in the present study, for rats with hippocampal residuals at the temporal pole to show slightly better performance than those with a residual septal pole is consistent with the functional connectivity of the temporal 40% of hippocampus spared in our experiments; this area, apart from featuring strong links to prefrontal cortex and subcortical sites (see Figure 1A), has some connectivity to the dorsolateral and adjacent intermediate domain of the entorhinal cortex [33], and cells at the septal end of this area show relatively accurate place-related firing [38].

### The Septal Pole of the Hippocampus Can Rapidly Form Place Representations, Even Though It Cannot Sustain Task Performance

Although rats with only the septal pole of the hippocampus were markedly impaired on the behavioral tests requiring rapid place learning, their residual hippocampal circuitry exhibited intact entorhinal-hippocampal plasticity and could rapidly, within one exposure to a novel environment, form accurate and stable place-related firing in CA1 pyramidal cells. Our electrophysiological findings support the idea [39,57] that residual hippocampal circuitry at the septal pole, due to connectivity to the dorsolateral domain of the entorhinal cortex, can rapidly form and maintain accurate place representations, even in the absence of the rest of the hippocampus. However, such representations on their own are not enough to sustain task performance based on rapid place learning, as revealed by the poor performance of rats with only the septal hippocampal pole intact. The deficits in navigational performance could reflect that larger-scale place representations, normally provided by neurons in more temporal parts of the hippocampus, are required for route planning [18,37,40]. However, a selective deficit in route planning, i.e., increased path lengths to reach the target area and relatively normal search preference for this area once it was reached, is not supported by our behavioral data. Rather, the marked performance deficits, despite an intact septal pole of the hippocampus maintaining accurate place-cell firing, highlight the importance of functional connectivity with prefrontal cortex and subcortical sites provided by the intermediate to temporal hippocampus.

Notably, apart from “pure” place codes, normal hippocampal firing can show additional characteristics, including some that are indicative of the animal's goals or motivation [7,13,106–116]. Candidate neuroanatomical substrates to confer motivational information to hippocampal neurons include subcortical afferents from dopaminergic midbrain neurons and amygdala that mainly target the intermediate to temporal hippocampus [29,30,32,117–119]. Therefore, removal of the temporal to intermediate hippocampus may disconnect place cells in the septal pole from motivational modulation; such disruption might also contribute to performance impairments on the rapid place-learning task. Interestingly, the reduced between-trials stability of the peak firing rate we found in CA1 place cells from septal residuals (experiment 4) may reflect a failure to accurately represent the procedural and motivational variables characterizing the recording trials (e.g., the rats had to perform a random-foraging task to receive food reward), given the ample evidence that the firing rate of place cells normally reflects such information [7,107–110,114–116].

### From Place Memory to Task Performance: Functional Integration in the Intermediate Hippocampus

Rats with a residual intermediate hippocampus performed better than all other partial lesion groups on all tests requiring rapid, one-trial, place learning. Remarkably, they generally performed as accurately and efficiently as the sham-operated control group. The exception to this was the retest on the rapid place-learning task in experiment 2, when rats with a residual intermediate hippocampus were performing slightly worse than control rats, though still better than all other lesion groups. This deficit was likely related to interference from the preceding incremental training to one location. Possibly, throughout incremental training, hippocampal cells become increasingly recruited in the representation of the target location [111], so that on a subsequent retest requiring rapid learning of a novel location, a reduced hippocampal circuit might have insufficient encoding capacity. Thus, it is important to note that even though a residual intermediate hippocampus can sustain performance based on rapid place learning, the septal and temporal tips are not redundant, but processing in these regions may be required to sustain normal behavior in more challenging situations. Nevertheless, the importance of the intermediate hippocampus for performance based on rapid place learning was further revealed by the finding that lesions removing this region, but sparing both the septal and temporal tip, completely abolished performance on the one-trial place-learning task, similar to complete hippocampal lesions.

The intermediate hippocampus combines connectivity to the dorsolateral domain of the entorhinal cortex, where visuospatial information is represented at a fine scale by grid cells with fine-grained firing patterns—and consistently relatively accurate place-related firing can be recorded from the intermediate hippocampus [25,36,38]—with connectivity to prefrontal cortex and subcortical sites, including nucleus accumbens and mediodorsal striatum (see Figure 1A). These connections are partially overlapping. In addition, extensive intrahippocampal projections [28,76–78], which mediate excitatory transmission [120,121], and synchronous neuronal activity [122] are found along the whole longitudinal extent of the intermediate hippocampus. Whereas the septal and temporal tip of the hippocampus, as spared in experiment 5, should together also posses the two complementary sets of connectivity (see Figure 1A), there are virtually no efficient connectional routes between the septal and temporal tip, except for those that involve steps in the intermediate region. Thus, the intermediate hippocampus anatomically and physiologically integrates two sets of functional links, namely to precise visuospatial processing, through the dorsolateral domain of the entorhinal cortex, and to behavioral control, through prefrontal cortex and subcortical sites, and our present data suggest that such integration is critical for the translation of rapid place learning into adaptive behavior.

Whereas the present study highlights that the intermediate hippocampus is critical for the translation of rapid place encoding into behavior, previous functional imaging studies in humans and mice have suggested that the intermediate hippocampus also plays a key role in the encoding and retrieval of multimodal associative memory [123–125]. More specifically, information flow through the middle or intermediate hippocampus may mediate the binding or integration of different sensory modalities that, due to the topography of the relevant neocortical connections, are initially represented at distant septotemporal levels. This view cannot readily account for the importance of the intermediate hippocampus for performance based on rapid place learning, as our place-cell recordings indicate that the relevant place encoding and retrieval can be sustained by the septal pole without contributions by other parts of the hippocampus. However, it also emphasizes the integrative properties of the intermediate hippocampus. Overall, an intriguing picture is thus emerging of the intermediate hippocampus as a key substrate for functional integration along the longitudinal axis.

## Materials and Methods

### Rats.

One hundred twenty-four adult male Lister-Hooded rats (Charles River) were used for the behavioral experiments (experiments 1, 2, and 5), 13 for the field-potential (experiment 3), and seven for the place-cell recordings (experiment 4). They were housed in groups of one to four in a temperature-controlled (20–23 °C) and humidity-controlled (40%–55%) room with an artificial light:dark cycle (lights on: 7 am to 7 pm). Rats had free access to food and water. Only for the place-cell experiments were rats fed a restricted diet to maintain them at approximately 90% of their free-feeding weight, so that they were motivated to forage in the recording arena. Rats weighed 250–350 g and were 10–14 wk old at surgery. Before the start of experiments, all rats were habituated to handling by the experimenters. Experimental procedures were conducted during the light phase of the cycle as far as possible. Home Office regulations for animal experimentation were followed (Project Licence No 60/2484).

### Surgery.

Partial or complete fiber-sparing lesions of the hippocampus were made under halothane or isoflurane anesthesia using bilateral stereotaxic microinjections of the neurotoxin ibotenic acid (Sigma; 10 mg/ml in 0.1 M phosphate-buffered saline) through a 1-μl SGE syringe (26 ga, 0.47-mm-diameter needle; WPI), as described in detail elsewhere [68], except that injection coordinates and volumes were adapted for the partial lesions to spare approximately 40% of hippocampal volume at different septotemporal levels. Partial or complete hippocampal lesions were achieved by injections of 0.05–0.10 μl at 7–13 sites in each hemisphere (see Table S2). For sham lesions, the empty injection needle was lowered into the neocortex at as many sites as required for the complete or partial hippocampal lesions, with the intention to induce comparable mechanical damage to the cortex.

For the place-cell experiments, sham lesions or partial lesions intended to spare the septal 40% of hippocampus were combined with the implantation of one or two sets of four tetrodes (four twisted, 17-μm polyimide-coated Pt-Ir[9:1] wires) mounted in an inner cannula (28 ga) connected to a light-weight microdrive (Axona); in addition, two rats without additional surgical treatment were implanted through small skull trepanations. The tetrodes were aimed above the CA1 layer in the septal pole of the hippocampus (bregma and lambda horizontally aligned, 3.5 mm posterior and 2.2–2.5 mm lateral from bregma, and 2.00 mm below dura). An outer protecting cannula (18 ga) on the microdrive was then lowered down to the dura, before Vaseline was applied around the cannula, and the trepanations above the lesions were filled with gauze for protection. The microdrive was secured to the skull using small screws, one of which served as electrical ground, and dental cement.

### Watermaze experiments (experiments 1, 2, 5).

*Watermaze and general procedures.* An open-field watermaze, 2 m in diameter and filled with water at 25 ± 1 °C made opaque by the addition of 200 μl of latex solution, was located in a well-lit room containing prominent visual cues at variable distance from the pool and visible from the water surface, so as to be used by the rats for orientation.

To start a trial, rats were placed into the water, facing the pool walls, at one of four start positions (north [N], east [E], south [S], and west [W]). They could escape on a single platform, 12 cm in diameter and hidden from the animals' sight 1–2 cm below the water surface. The “Atlantis platform” was used, which can be withheld at >30 cm below the water surface for a predetermined time, by a computer-controlled electromagnet, before being raised to its normal position [126]. This enabled rewarded probe trials during which the rats' search preference is first monitored for 60 s, and the platform is then made available to reinforce spatially focused searching. Our paradigms involved testing with different platform locations on an inner ring (0.8-m diameter) or outer ring (1.4 m) concentric with the pool (Figure S9). Every trial ended with the rat sitting for 30 s on the platform before being returned to its cage. If an animal failed to reach the platform within 120 s, it was guided there by the experimenter.

The rats' behavior was monitored by an overhead video camera connected to a video recorder and a computer with WaterMaze software (Actimetrics) in an adjacent control room. The software aided collection of the following measures: latency and path length to reach the platform location, swim speed, and the percentage time spent in the correct zone (see *Zone analysis of search preference* below).

*Rapid place-learning task.* A modification of the delayed-matching-to-place task [70] was used, in which trial 2 of a day was occasionally run as a probe trial. Rats received four trials a day. The platform was hidden in a novel location on trial 1 of each day and then remained in this place for trials 2–4, on which rats could use rapidly encoded place memory to reach the escape platform efficiently. All four start positions were used daily in an arbitrary sequence, to discourage egocentric strategies. Analysis focused on trial 2 of each day, on which performance relied on place memory encoded within a single trial, whereas trial 3 and 4 were run to reinforce the win-stay rule of the task. Trials 1 and 2 were 10–30 s or 20 min apart (i.e., one-trial place memory on trial 2 was assessed at two different retention delays), whereas the delay was 10–30 s between the other trials. Search preference in the correct location on probe trials has long been recognized as the most reliable measure of place memory in the watermaze, whereas latency measurements are highly dependent on chance, and may be reduced efficiently through systematic search strategies and the use of single beacon cues and through a coarse estimate of position and sense of direction mediated partly by extrahippocampal structures [71]. Therefore, as an important modification of the original task, which purely relied on latency measurements [70], trial 2 was occasionally run as rewarded probe trial to measure the rats' search preference for the zone containing the platform location.

*Incremental place-learning task.* Rats were trained to a constant platform location with eight trials a day, ca. 10 min (±3 min) apart, for 5 d (i.e., 40 trials altogether). Trials 2 and 6 of each day were run as rewarded probe trials. Start positions changed between trials, to discourage egocentric strategies. An additional rewarded probe trial was run on day 6, to assess incrementally acquired memory unconfounded by within-day learning.

*Zone analysis of search preference.* Search preference for the vicinity of the platform location on probe trials was assessed as follows: Eight 40-cm-diameter “virtual” zones, one of which (the “correct zone”) was concentric with the platform location, were defined on the inner and outer ring of the pool, so that the zones were nonoverlapping, evenly spaced, and symmetrically arranged (Figure S9). The time spent in each of these eight zones during the 60-s probe trial was determined automatically using the WaterMaze software. From these measures the “percentage of time spent in the correct zone” was calculated as: (time in correct zone/time in all eight zones) × 100%. By chance, i.e., during random swimming, this value should be: 100%/8 = 12.5%, whereas higher values indicate a search preference for the correct zone. The fact that the comparison zones were located on the inner and outer rings was critical to unequivocally identify search preference due to one-trial place memory on the rapid place-learning task: throughout training, platform locations were always on the inner or outer ring, and hence, a search preference for a zone in this area as compared to a zone elsewhere on the pool surface might have merely reflected procedural and incremental learning.

*Experiment 1.* Three groups with partial hippocampal lesions sparing approximately 40% of hippocampal volume at the septal or temporal pole, or in the intermediate region, a sham-lesioned control group, and a group with complete hippocampal lesions were tested on the rapid place-learning task. Experiment 1 was run in five replications, each comprising 20 rats and always including sham-lesioned controls. Rats were pretrained on the rapid place-learning task for 8 d before the lesion surgery. After pretraining, rats were divided into groups, which were matched for all analyzed performance measures as far as possible, to receive the different lesions within 5 d. After at least 1 wk of recovery, 8 d of postsurgical testing on the rapid place-learning task commenced. Two sets of eight different platform locations (one novel location per day) were used during pretraining and postsurgical testing, with the eight locations evenly distributed across the pool (Figure S9). During both pretraining and postsurgical testing, the two delays between trials 1 and 2 (10–30 s or 20 min) were used equally often, and on days 4 and 8, trial 2 was run as probe trial. Two delays and two platform locations were used each day in all four possible combinations, so that the different delays were paired with the different locations in a counterbalanced manner across days with and without probe trials. The allocation of the different lesion groups to the different sequences of platform-delay combinations was counterbalanced.

*Experiment 2.* The lesion groups from experiment 1 were further tested on the incremental place-learning task, commencing 1–4 d after testing on the rapid place-learning task was completed. To avoid that results were confounded by the properties of one particular platform location (rats tend to find some locations more easily than others), two platform locations were used, counterbalanced between groups, in distant parts of the pool. One location was on the outer ring in the N, the other one on the inner ring in the SW (Figure S9).

Two days after testing on the incremental place-learning task, rats were retested for 1 d on the rapid place-learning task with a delay of 20 min between trials 1 and 2. Rats trained to the location on the outer ring (N) were switched to the location on the inner ring (SW) and vice versa.

*Experiment 5.* Rats with lesions to the intermediate hippocampus, sparing approximately 20% at the septal and temporal tips, and a sham-lesioned control group were compared on the rapid place-learning task. The experiment was run in one replication including 12 rats in each group. Pre- and postsurgical testing was as described for experiment 1.

### Evoked-potential recordings (experiment 3).

Field potentials from the dentate-gyrus granule cell layer in the septal hippocampus evoked by stimulation of the perforant path were recorded from anesthetized rats (urethane, 1.5 g/kg intraperitoneally) that had received sham lesions or partial lesions sparing the septal pole, followed by at least 1 wk of recovery. Electrode coordinates and procedures were similar to previous experiments [82]. The slope of the initial rising part (2.0–2.6 ms or 2.2–2.8 ms after stimulation) and the population spike amplitude were used as measures of the evoked responses. After positioning the electrodes, low-frequency baseline stimulation (biphasic 0.1-ms pulses, 0.05 Hz) at 0.5 mA was applied until responses were stable. Recurrent feedback inhibition was tested by applying paired pulses, 20 ms apart, at 0.5 mA (15 pairs, 10 s apart) [75]. To determine input–output curves, stimulation intensity was increased from 0.1 to 1 mA in 0.1-mA steps (three stimulations per step, 0.1 Hz). To measure LTP, stimulus intensity was adjusted to obtain responses whose slope was approximately 50% of the slope maximum in the input–output curve, and low-frequency baseline stimulation (biphasic 0.1-ms pulses, 0.05 Hz) was applied for at least 20 min before tetanization. A tetanus consisted of three trains of 50 biphasic 0.2-ms pulses at 250 Hz with 60 s between trains (overall 2 min). Following the tetanus, low-frequency baseline stimulation continued for an additional 60 min. At the end of the experiments, the locations of the electrode tips were marked by a 10-mA, 2-s biphasic pulse to the electrodes. The rats were then perfused and their brains further processed as described under Histology, below.

### Place-cell recordings (experiment 4).

Starting 4 to 7 d after tetrode implantation, rats were trained to forage in a 1-m × 1-m arena with a brown floor enclosed by four transparent Perspex walls (40-cm high) and placed in a room with many distal cues. Water-soaked Rice Krispies were continuously scattered across the arena by the experimenter from behind a black curtain, so that the food-deprived rats moved continuously within the whole open field. Tetrodes were advanced daily to the CA1 pyramidal cell layer, and the rats became familiar with the random-foraging task and the environment. Neurophysiological signals from the tetrodes were recorded in parallel to the rat's path using Axona systems (Axona). Once well-isolated hippocampal pyramidal cells were identified, experimental recordings began. Cells were recorded while the rats foraged for two ca. 15-min trials in the familiar environment (Fam) (Figure 5B); the trials were separated by typically 30 to 60 min, which the rat spent in a holding bucket and during which the arena was cleaned. Recordings in the familiar environment were repeated on subsequent days as the electrodes encountered new cells. In three rats (one lesioned, two control), cells were also recorded during exposure to a new environment (New) to examine the rapid formation of place-related firing patterns. For this purpose, the familiar arena was replaced by a new 0.64-m × 0.94-m arena with a black floor and 3-cm lips, and two prominent distal visual cues were changed. The rats foraged during four ca. 15-min trials (Fam-New-New-Fam), separated by ca. 10 min in the bucket (Figure 5C). Spike sorting and subsequent detailed analysis of the firing characteristics and patterns of well-isolated pyramidal cells were performed offline with dedicated software (for further details, see Text S1, Supplementary Materials and Methods).

### Statistical analysis.

In the watermaze experiments (experiments 1, 2, and 5), ANOVA (with trials as within-subjects factor), followed by Fisher LSD post hoc tests, was used to analyze group differences. Paired two-tailed Student *t*-tests were used to compare latencies and path lengths on trials 1 and 2, as well as the percentage time in the correct zone with the chance level, in individual groups. In the field-potential study (experiment 3), ANOVA was used to analyze input–output curves. To analyze LTP, slopes were averaged in 5-min blocks and expressed as a percentage of the mean slope during the 20-min baseline recording. Paired two-tailed Student *t*-tests were used to demonstrate LTP, and unpaired two-tailed *t*-tests were used for group comparisons. In the single-unit experiment (experiment 4), unpaired two-tailed Student *t*-tests were used to compare measures between the two groups. Accepted level of significance was *p* < 0.05.

### Histology.

After completion of the experiments, rats were anesthetized with an overdose of Euthatal (Harlow) and perfused transcardially with saline, followed by 4% formaldehyde solution. Brains were extracted from the skull, postfixed in 4% formaldehyde solution for at least 24 h, and then egg-embedded as described elsewhere [68]. Coronal 30-μm sections were cut on a freezing microtome, and every fifth section—or, for verification of electrode locations, every section—through the hippocampus was mounted on gelatine-coated slides and stained with cresyl-violet. For documentation, digital photographs were taken from exemplar brains.

### Lesion analysis and three-dimensional visualization of spared hippocampal tissue.

To measure the relative volume of spared hippocampal tissue, the intact hippocampal area (dentate gyrus, CA1–3 fields) on every fifth section was outlined at 10× magnification for each brain and measured using a binocular connected via a digital camera to a computer running Leica Q Win software. These areas were summed up for each brain, and the mean hippocampal area was calculated for each group. The relative hippocampal volume was calculated by dividing the hippocampal areas in individual brains by the mean hippocampal area determined for the relevant sham-lesioned control group.

For the three-dimensional visualization and comparison of the distinct chunks of spared hippocampus in the different groups, Neurolucida and NeuroExplorer software (MicroBrightField Europe) on a computer connected to a microscope via a digital camera were used to prepare three-dimensional reconstructions of the hippocampus (dentate gyrus, CA1–3) together with the brain silhouette from the coronal sections (cut 150 μm apart) of exemplar brains.

## Supporting Information

Figure S1 Experiment 1: Pretraining Performance in the Five Prospective Surgical Groups at the Two Different Retention Delays between Trials 1 and 2 The prospective surgical groups, to receive different hippocampal lesions or sham surgery (see Figure 1C), were matched for: (A) percentage time (%time) in correct zone (mean ± SEM; chance level 12.5%, indicated by stippled line), when trial 2 was run as a probe on days 4 and 8; (B) latencies (mean ± SEM) to reach the platform, averaged across days (all relevant main effects: *F* < 1; interactions: *F* < 1.5, *p* > 0.14). At this stage of the experiment, there was no significant dependence of performance measures on the delays between trial 1 and 2 (all relevant main effects: *F* < 1.1, *p* > 0.30; interactions: *F* < 2.6, *p* > 0.05). In all groups, performance based on one-trial place memory was indicated by a %time in correct zone about twice as high as expected based on chance and a significant latency reduction from trial 1 to 2 (all *t* > 5.3, *p* < 0.0005).
(151 KB PDF)

Figure S2 Experiment 1: Postsurgical Performance on the Rapid Place-Learning Task with a 15- to 30-s or a 20-min Delay between Trials 1 and 2 (A) Percentage time (%time) in the correct zone (mean ± SEM; chance level 12.5%, indicated by stippled line) when trial 2 was conducted as a probe trial either 15–30 s or 20 min after trial 1 (one probe per delay). Apart from the significant main effect of group (see main text), there was a significant main effect of delay (*F*_1,84_ = 6.10, *p* < 0.02), independent of group (interaction: *F*_4,84_ = 1.45, *p* > 0.2), indicating forgetting of one-trial place memory.
(B) Path lengths (mean ± SEM) to reach the platform, shown separately for the days with a trial 1 to trial 2 delay of 10–30 s and 20 min (4 d at each delay). Apart from the trial-dependent group differences (see main text), there was a main effect of delay (*F*_1,84_ = 8.32, *p* < 0.005), but only a strong tendency for this effect to be trial-dependent (*F*_3,252_ = 2.44, *p* = 0.06); the effects and interactions involving delay were group independent (F < 1.63, *p* > 0.17). That percentage time in correct zone during presurgical testing (Figure S1) did not show delay-dependence may reflect that one of the probe trials was run on day 4, when performance had not quite yet reached asymptotic levels.
(241 KB PDF)

Figure S3 Experiment 2: Performance on the Incremental Place-Learning Task across All Days and Trials (A) Path lengths (mean ± SEM) to reach the platform on the eight daily trials (T1–T8) of days 1–5 and on the single trial on day 6.
(B) Percentage time (%time) in correct zone (mean ± SEM; chance level 12.5%, indicated by stippled line) on the trials that were run as probes (T2 and T6 from days 1 to 5 and T1 on day 6).
The group differences in both measures decreased across trials within days and, especially, between days. This was supported by highly significant group × trial interactions (path lengths: *F*_160,3360_ = 1.69, *p* < 0.0001; search preference: *F*_40,840_ = 2.28, *p* < 0.0001).
(255 KB PDF)

Figure S4 Experiment 2: Path Lengths and Savings during Retesting on the Rapid Place-Learning Task Only sham-operated rats and the group with hippocampal lesions sparing the intermediate region showed a significant reduction in path lengths from trial 1 to 2 (*t* > 3.54, *p* < 0.001; all other *t* < 1.23, *p* > 0.24), i.e., savings (group × trial interaction for path lengths: *F*_12,201_ = 3.91, *p* < 0.0001; main effect of group on savings: *F*_4,67_ = 6.28, *p* < 0.0003). Group differences: an asterisk (*) indicates different from sham-operated group and group with only the intermediate hippocampus spared (*p* < 0.015); a number sign (#) indicates different from group with only the intermediate hippocampus spared (*p* < 0.03).
(153 KB PDF)

Figure S5 Experiment 4: Firing-Rate and Path Maps for All Cells Recorded from Septal CA1 during Two Successive Recording Trials in the Familiar Environment (Fam) Recordings from rats with hippocampal residuals at the septal pole (A) and from rats with an intact hippocampus (B) are shown. The color-coded firing-rate maps and, below, the location of spikes (red) on the rat's trajectory (gray) are shown. The peak firing rate for each trial is indicated in red numbers above, and the correlation between the firing-rate maps for the two successive trials is shown in black below. Quantitative measures of the firing properties of all cells in both groups of rats are summarized in Table S1.
(2.6 MB PDF)

Figure S6 Experiment 4: Firing-Rate and Path Maps for All Cells Recorded from Septal CA1 during a Succession of 15-min Trials in the Familiar Environment (Fam) and the New Environment (New) Recordings from hippocampal residuals at the septal pole, left, and from an intact hippocampus, right, are shown. The color-coded firing-rate maps and, below, the location of spikes (red) on the rat's trajectory (gray) are shown. All firing rate maps are scaled to the same maximum firing rate, equal to the maximum of all four trials and indicated in red to the right; correlations between the firing rate maps for different trials are indicated in black below.
(1.13 MB PDF)

Figure S7 Experiment 5: Presurgical Training on the Rapid Place-Learning Task and Performance Matching (A) Latencies (mean ± SEM) to reach the platform on the four daily trials across the 8 d of presurgical training (data are shown for the 23 rats that were included in the final analysis). Data were collapsed for the two different delays between trial 1 and 2 (15–30 s or 20 min), which were used equally often and for half of the rats each on every day. On days 4 and 8, trial 2 was run as probe.
(B and C) The prospective surgical groups, to receive sham surgery (*n* = 12) or lesions to the intermediate hippocampus (*n* = 11), were matched for: (B) percentage time (%time) in correct zone (mean ± SEM; chance level 12.5%, indicated by stippled line), when trial 2 was run as a probe on days 4 and 8; (C) latencies (mean ± SEM) to reach the platform, averaged across days (all relevant main effects: *F* < 1; interactions: *F* < 2.2, *p* > 0.15). There was no significant dependence of performance measures on the delays between trial 1 and 2 (all relevant main effects: *F* < 1; interactions: *F* < 2.0, *p* > 0.17). In both groups, performance based on one-trial place memory was indicated by a significant latency reduction from trial 1 to 2, and a percentage time in the correct zone about twice as high as expected based on chance (*t*_11_ > 4.28, *p* < 0.002).
(195.31 KB PDF)

Figure S8 Experiment 5: Postsurgical Performance on the Rapid Place-Learning Task with a 15- to 30-s or a 20-min Delay between Trials 1 and 2 (A) Percentage time (%time) in the correct zone (mean ± SEM; chance level 12.5%, indicated by stippled line) when trial 2 was conducted as a probe trial either 15–30 s or 20 min after trial 1 (one probe per delay). There was only a significant main effect of group (see main text), but no main effect of delay nor an interaction group × delay (both *F*_1,22_ < 1).
(B) Path lengths (mean ± SEM) to reach the platform, shown separately for the days with a trial 1 to trial 2 delay of 10–30 s and 20 min (4 d at each delay).
Apart from the trial-dependent group differences (see main text), there were no significant main effects or interactions (all *F* < 2.3, *p* > 0.09).
(155 KB PDF)

Figure S9 Platform Locations Used During the Different Stages of Behavioral Testing and the Surrounding Zones Used for the Analysis of Search Preference (96 KB PDF)

Table S1 Firing Properties of Complex Spike Cells in Septal CA1 Recorded from Control Rats with an Intact Hippocampus and from Rats with Partial Hippocampal Lesions Sparing Only the Septal Pole (105 KB DOC)

Table S2 Stereotaxic Coordinates (in Millimeters from Bregma) and Injection Volumes for the Hippocampal Ibotenic Acid Injections in the Different Lesion Groups (53 KB DOC)

Text S1 The Supporting Text containing Supplementary Results, Supplementary Discussion, Supplementary Materials and Methods, and Supplementary References (86 KB DOC)

Video S1 Intact Hippocampus Videos S1–S5 show rotations of the 3D reconstructions depicted in Figures 1C and 6B; please note that, different from the figures, hippocampal residuals are not overlaid on an intact hippocampus.
(2.24 MB MOV)

Video S2 Hippocampal Residual in the Intermediate Region (2.20 MB MOV)

Video S3 Hippocampal Residual at the Temporal Pole (2.44 MB MOV)

Video S4 Hippocampal Residual at the Septal Pole (2.66 MB MOV)

Video S5 Hippocampal Residuals at the Temporal and Septal Tip Following Selective Lesion of the Intermediate Region (2.92 MB MOV)

## Abbreviations

LTP: long-term potentiation
SEM: standard error of the mean
