# Supplementary material for: From Rapid Place Learning to Behavioral Performance: A Key Role for the Intermediate Hippocampus
Source: PLoS Biol. 2009 Apr 21;7(4):e1000089. doi: 10.1371/journal.pbio.1000089 (PMC2671558; doi:10.1371/journal.pbio.1000089)
Supplement: Supplementary file 1 [file pbio.1000089.sg001.pdf]

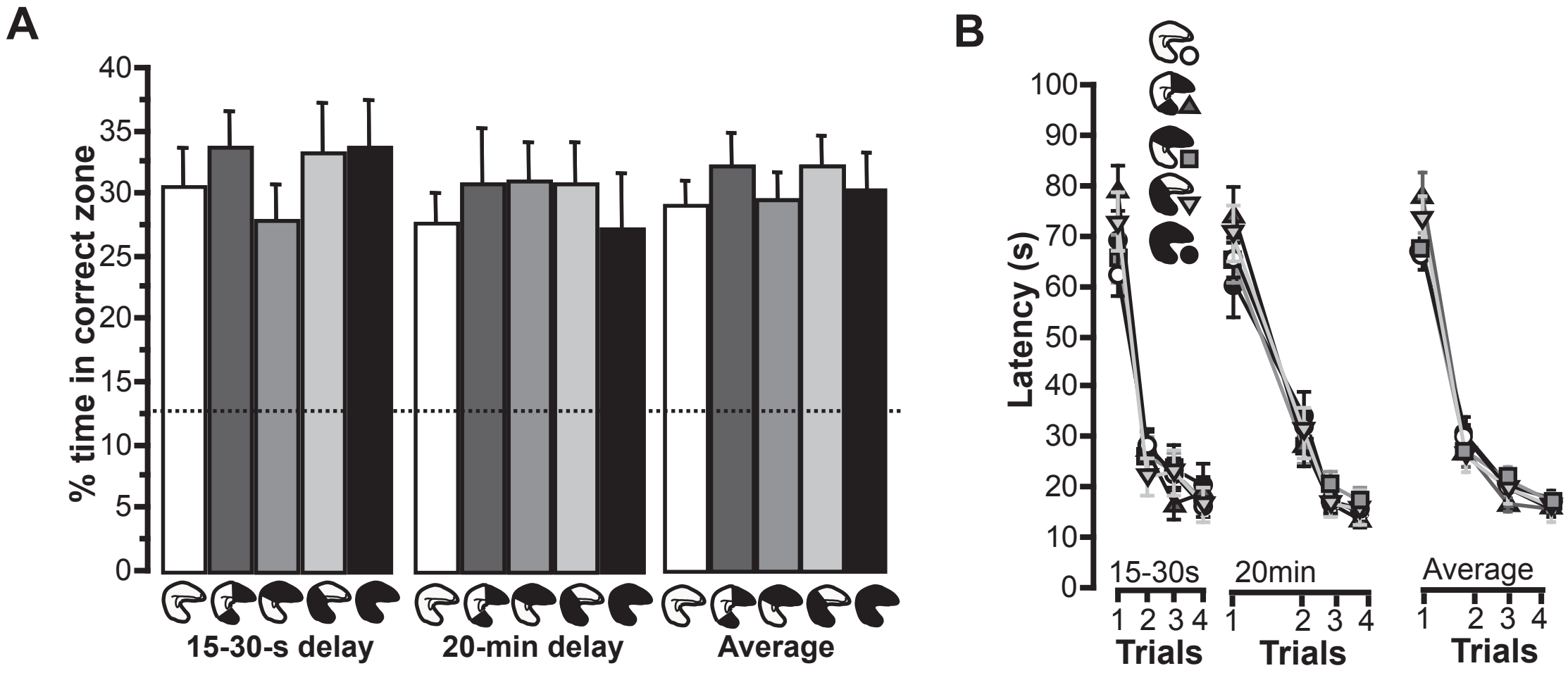

A
% time in correct zone
40
35
30
25
20
15
10
5
0
15-30-s delay
20-min delay
Average
B
Latency (s)
100
90
80
70
60
50
40
30
20
10
0
15-30s
20min
Average
1 2 3 4
Trials
