# Supplementary material for: From Rapid Place Learning to Behavioral Performance: A Key Role for the Intermediate Hippocampus
Source: PLoS Biol. 2009 Apr 21;7(4):e1000089. doi: 10.1371/journal.pbio.1000089 (PMC2671558; doi:10.1371/journal.pbio.1000089)
Supplement: Supplementary file 3 [file pbio.1000089.sg003.pdf]

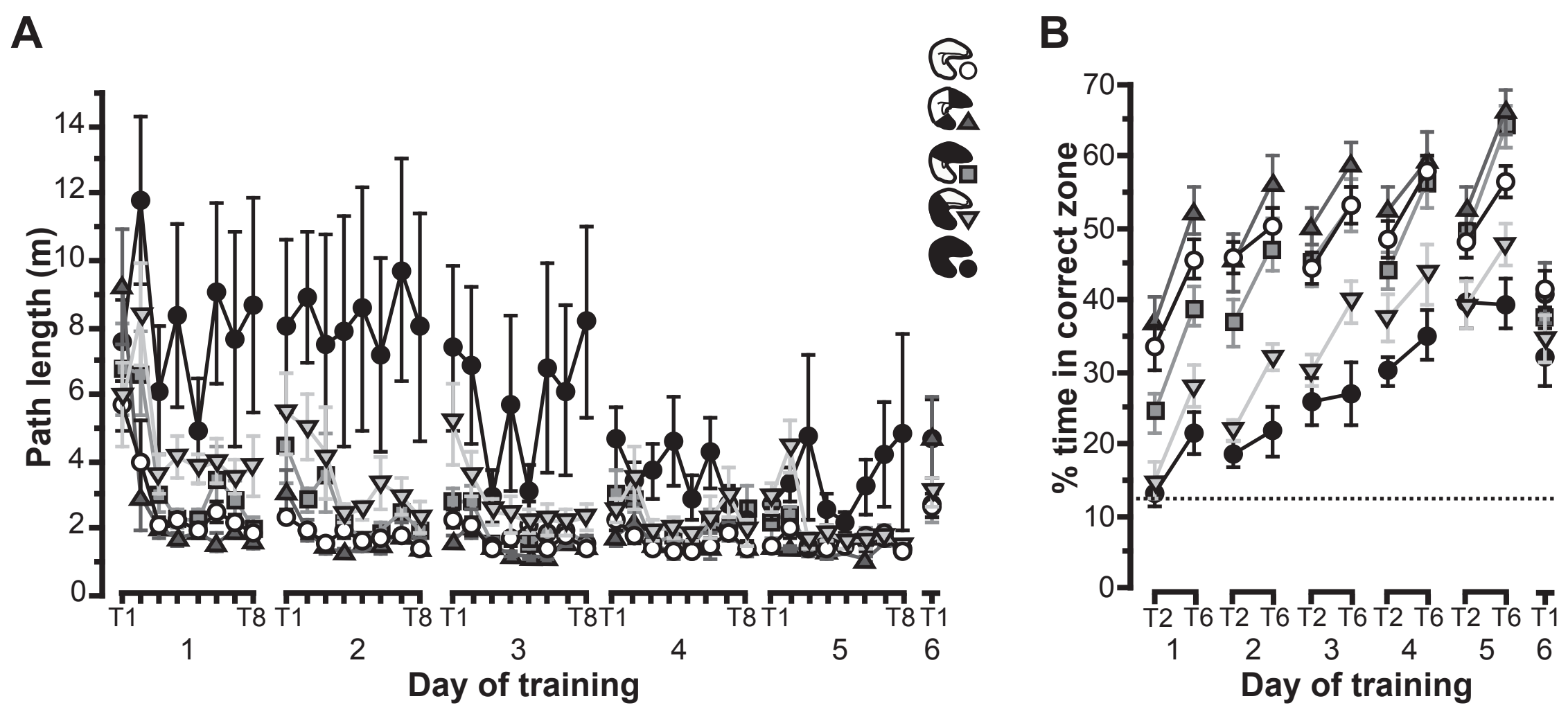

A
B
Path length (m)
0
2
4
6
8
10
12
14
T1
T8
1
2
3
4
5
6
Day of training
% time in correct zone
0
10
20
30
40
50
60
70
T2
T6
