# Supplementary material for: From Rapid Place Learning to Behavioral Performance: A Key Role for the Intermediate Hippocampus
Source: PLoS Biol. 2009 Apr 21;7(4):e1000089. doi: 10.1371/journal.pbio.1000089 (PMC2671558; doi:10.1371/journal.pbio.1000089)
Supplement: Supplementary file 5 [file pbio.1000089.sg005.pdf]

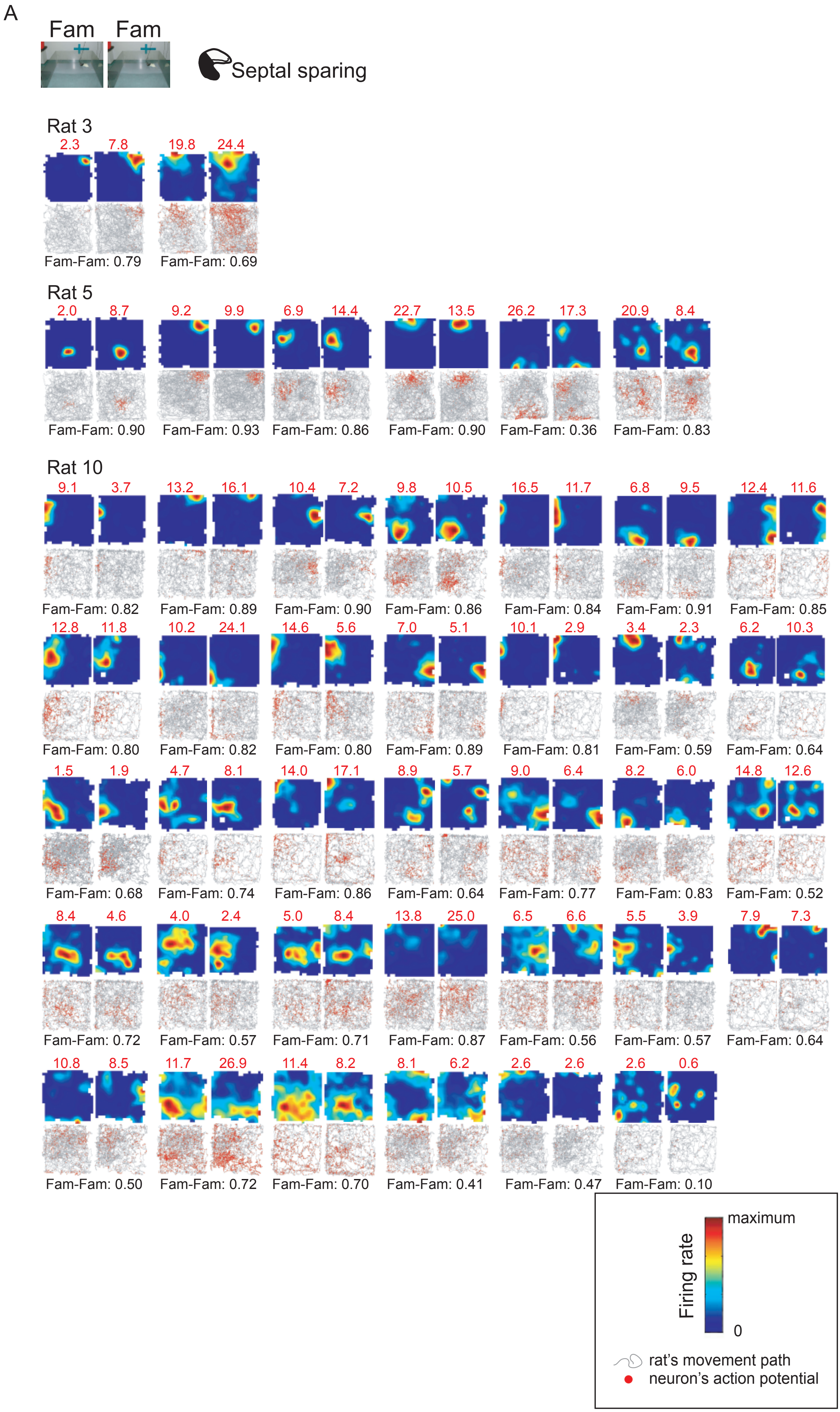
A
Fam Fam
Septal sparing
Rat 3
2.3 7.8 19.8 24.4
Fam-Fam: 0.79 Fam-Fam: 0.69
Rat 5
2.0 8.7 9.2 9.9 6.9 14.4 22.7 13.5 26.2 17.3 20.9 8.4
Fam-Fam: 0.90 Fam-Fam: 0.93 Fam-Fam: 0.86 Fam-Fam: 0.90 Fam-Fam: 0.36 Fam-Fam: 0.83
Rat 10
9.1 3.7 13.2 16.1 10.4 7.2 9.8 10.5 16.5 11.7 6.8 9.5 12.4 11.6
Fam-Fam: 0.82 Fam-Fam: 0.89 Fam-Fam: 0.90 Fam-Fam: 0.86 Fam-Fam: 0.84 Fam-Fam: 0.91 Fam-Fam: 0.85
12.8 11.8 10.2 24.1 14.6 5.6 7.0 5.1 10.1 2.9 3.4 2.3 6.2 10.3
Fam-Fam: 0.80 Fam-Fam: 0.82 Fam-Fam: 0.80 Fam-Fam: 0.89 Fam-Fam: 0.81 Fam-Fam: 0.59 Fam-Fam: 0.64
1.5 1.9 4.7 8.1 14.0 17.1 8.9 5.7 9.0 6.4 8.2 6.0 14.8 12.6
Fam-Fam: 0.68 Fam-Fam: 0.74 Fam-Fam: 0.86 Fam-Fam: 0.64 Fam-Fam: 0.77 Fam-Fam: 0.83 Fam-Fam: 0.52
8.4 4.6 4.0 2.4 5.0 8.4 13.8 25.0 6.5 6.6 5.5 3.9 7.9 7.3
Fam-Fam: 0.72 Fam-Fam: 0.57 Fam-Fam: 0.71 Fam-Fam: 0.87 Fam-Fam: 0.56 Fam-Fam: 0.57 Fam-Fam: 0.64
10.8 8.5 11.7 26.9 11.4 8.2 8.1 6.2 2.6 2.6 2.6 0.6
Fam-Fam: 0.50 Fam-Fam: 0.72 Fam-Fam: 0.70 Fam-Fam: 0.41 Fam-Fam: 0.47 Fam-Fam: 0.10
Firing rate
maximum
0
rat's movement path
neuron's action potential

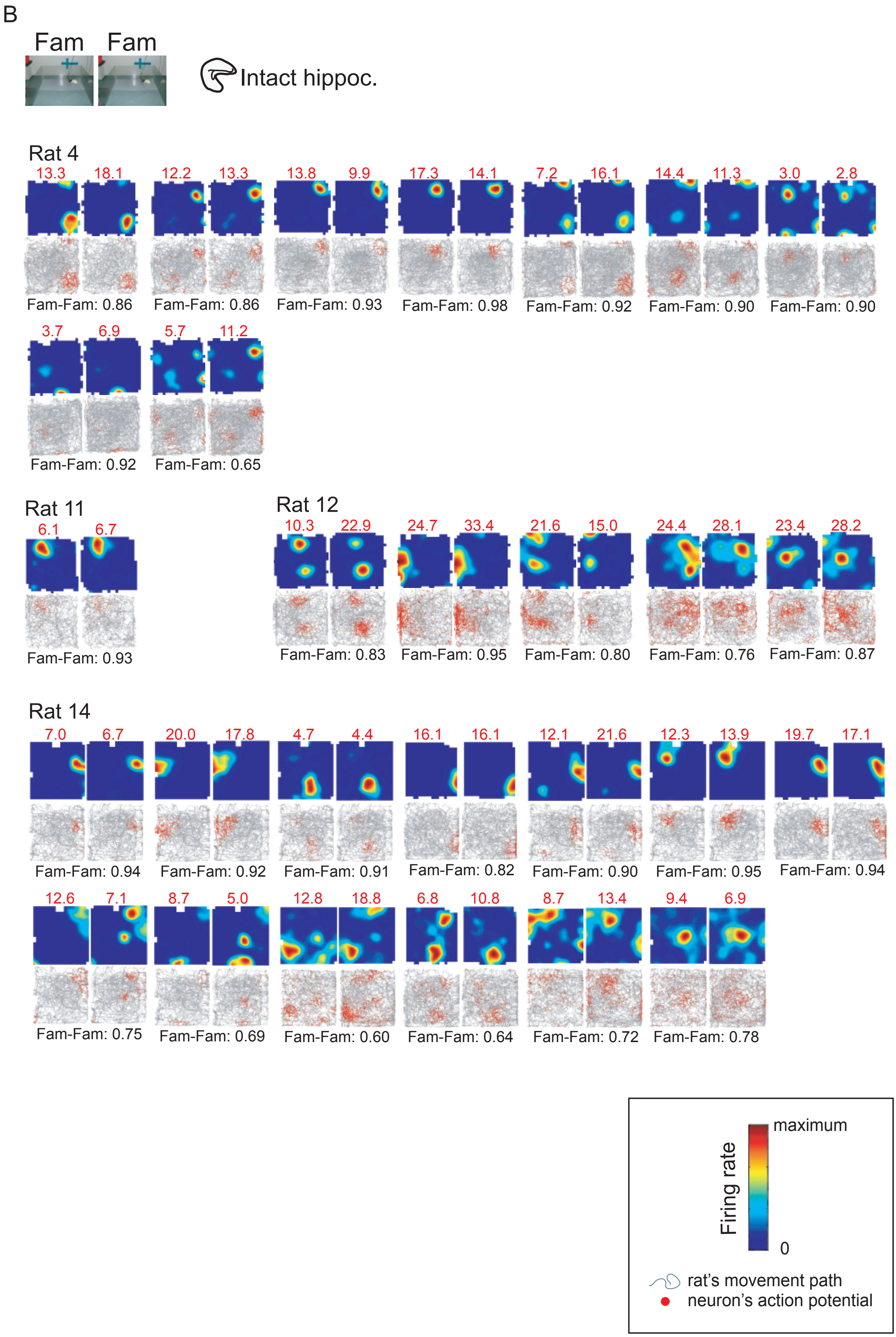
B
Fam Fam
Intact hippoc.
Rat 4
13.3 18.1 Fam-Fam: 0.86
12.2 13.3 Fam-Fam: 0.86
13.8 9.9 Fam-Fam: 0.93
17.3 14.1 Fam-Fam: 0.98
7.2 16.1 Fam-Fam: 0.92
14.4 11.3 Fam-Fam: 0.90
3.0 2.8 Fam-Fam: 0.90
3.7 6.9 Fam-Fam: 0.92
5.7 11.2 Fam-Fam: 0.65
Rat 11
6.1 6.7 Fam-Fam: 0.93
Rat 12
10.3 22.9 Fam-Fam: 0.83
24.7 33.4 Fam-Fam: 0.95
21.6 15.0 Fam-Fam: 0.80
24.4 28.1 Fam-Fam: 0.76
23.4 28.2 Fam-Fam: 0.87
Rat 14
7.0 6.7 Fam-Fam: 0.94
20.0 17.8 Fam-Fam: 0.92
4.7 4.4 Fam-Fam: 0.91
16.1 16.1 Fam-Fam: 0.82
12.1 21.6 Fam-Fam: 0.90
12.3 13.9 Fam-Fam: 0.95
19.7 17.1 Fam-Fam: 0.94
12.6 7.1 Fam-Fam: 0.75
8.7 5.0 Fam-Fam: 0.69
12.8 18.8 Fam-Fam: 0.60
6.8 10.8 Fam-Fam: 0.64
8.7 13.4 Fam-Fam: 0.72
9.4 6.9 Fam-Fam: 0.78
Firing rate
maximum
0
rat's movement path
neuron's action potential
