# Supplementary material for: From Rapid Place Learning to Behavioral Performance: A Key Role for the Intermediate Hippocampus
Source: PLoS Biol. 2009 Apr 21;7(4):e1000089. doi: 10.1371/journal.pbio.1000089 (PMC2671558; doi:10.1371/journal.pbio.1000089)
Supplement: Supplementary file 6 [file pbio.1000089.sg006.pdf]

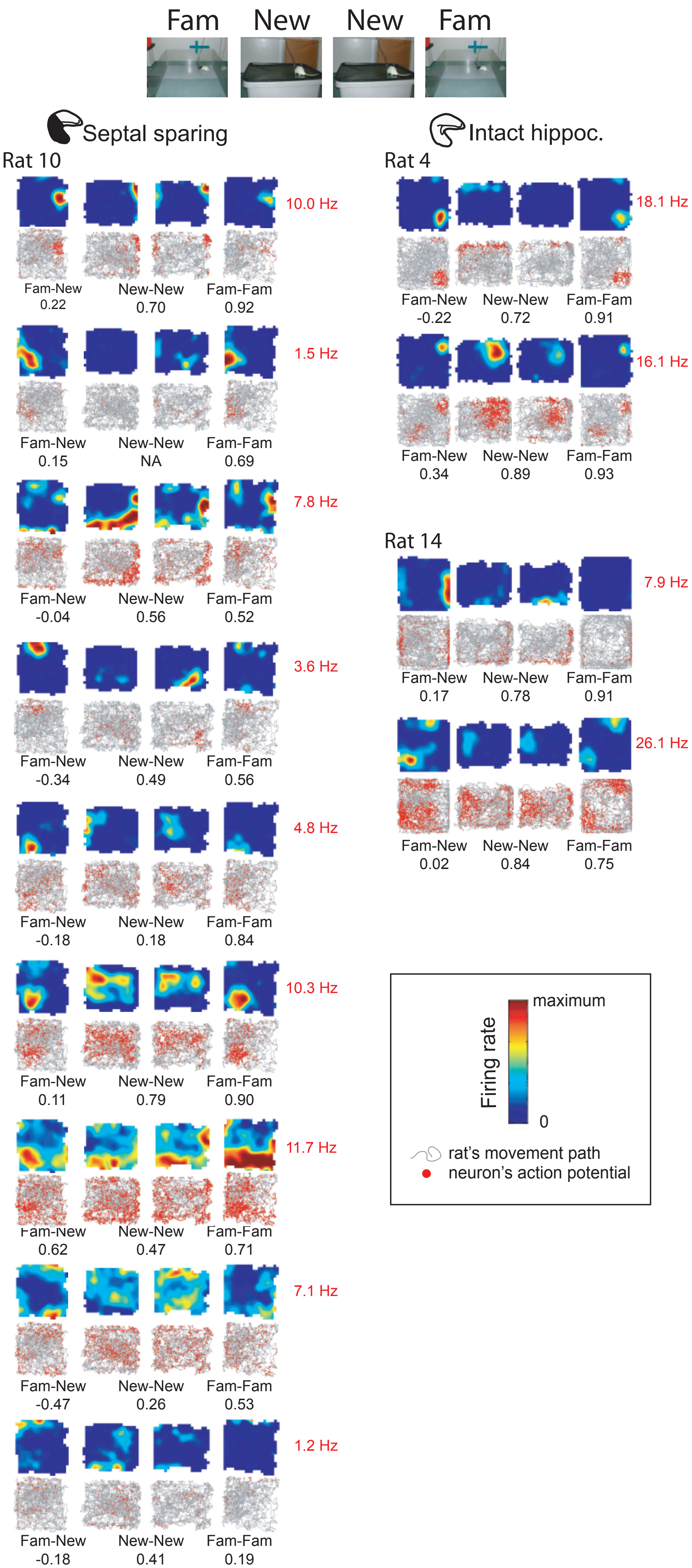

Fam
New
New
Fam
Septal sparing
Intact hippoc.
Rat 10
10.0 Hz
Fam-New 0.22
New-New 0.70
Fam-Fam 0.92
1.5 Hz
Fam-New 0.15
New-New NA
Fam-Fam 0.69
7.8 Hz
Fam-New -0.04
New-New 0.56
Fam-Fam 0.52
3.6 Hz
Fam-New -0.34
New-New 0.49
Fam-Fam 0.56
4.8 Hz
Fam-New -0.18
New-New 0.18
Fam-Fam 0.84
10.3 Hz
Fam-New 0.11
New-New 0.79
Fam-Fam 0.90
11.7 Hz
Fam-New 0.62
New-New 0.47
Fam-Fam 0.71
7.1 Hz
Fam-New -0.47
New-New 0.26
Fam-Fam 0.53
1.2 Hz
Fam-New -0.18
New-New 0.41
Fam-Fam 0.19
Rat 4
18.1 Hz
Fam-New -0.22
New-New 0.72
Fam-Fam 0.91
16.1 Hz
Fam-New 0.34
New-New 0.89
Fam-Fam 0.93
Rat 14
7.9 Hz
Fam-New 0.17
New-New 0.78
Fam-Fam 0.91
26.1 Hz
Fam-New 0.02
New-New 0.84
Fam-Fam 0.75
Firing rate
maximum
0
rat's movement path
neuron's action potential
