# Supplementary material for: From Rapid Place Learning to Behavioral Performance: A Key Role for the Intermediate Hippocampus
Source: PLoS Biol. 2009 Apr 21;7(4):e1000089. doi: 10.1371/journal.pbio.1000089 (PMC2671558; doi:10.1371/journal.pbio.1000089)
Supplement: Supplementary file 7 [file pbio.1000089.sg007.pdf]

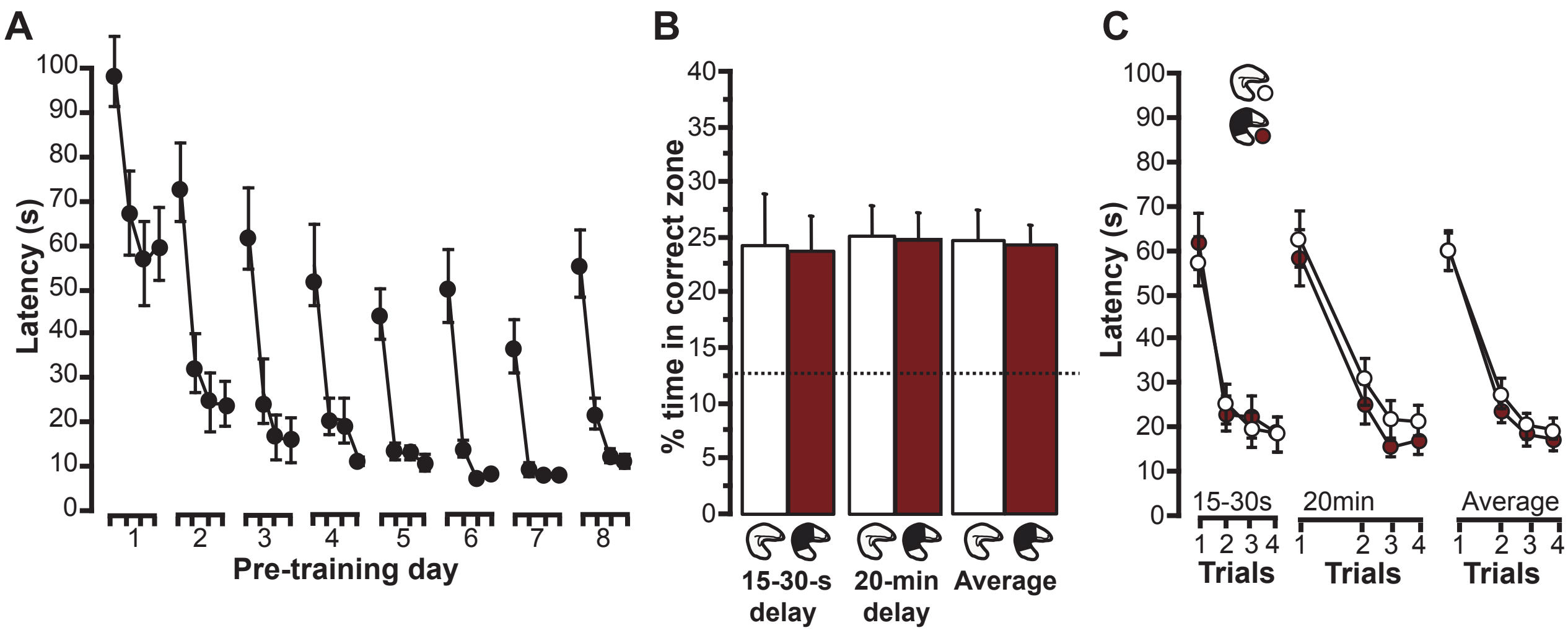

A
Latency (s)
Pre-training day
B
% time in correct zone
15-30-s delay
20-min delay
Average
C
Latency (s)
15-30s
20min
Average
Trials
Trials
Trials
