# Supplementary material for: From Rapid Place Learning to Behavioral Performance: A Key Role for the Intermediate Hippocampus
Source: PLoS Biol. 2009 Apr 21;7(4):e1000089. doi: 10.1371/journal.pbio.1000089 (PMC2671558; doi:10.1371/journal.pbio.1000089)
Supplement: Supplementary file 8 [file pbio.1000089.sg008.pdf]

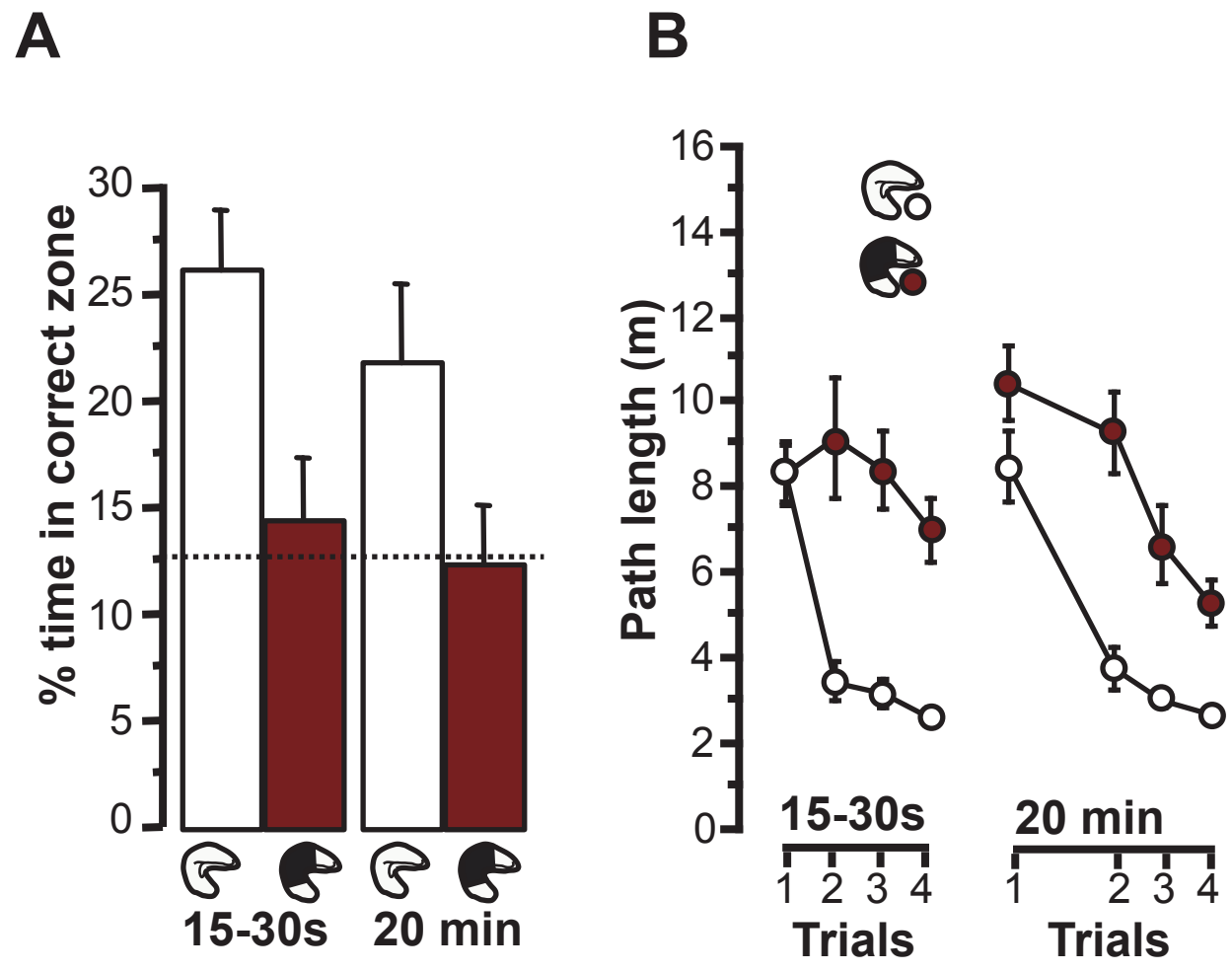
A
B
% time in correct zone
0
5
10
15
20
25
30
15-30s
20 min
Path length (m)
0
2
4
6
8
10
12
14
16
15-30s
20 min
1 2 3 4
1 2 3 4
Trials
Trials
