# Supplementary material for: From Rapid Place Learning to Behavioral Performance: A Key Role for the Intermediate Hippocampus
Source: PLoS Biol. 2009 Apr 21;7(4):e1000089. doi: 10.1371/journal.pbio.1000089 (PMC2671558; doi:10.1371/journal.pbio.1000089)
Supplement: Supplementary file 9 [file pbio.1000089.sg009.pdf]

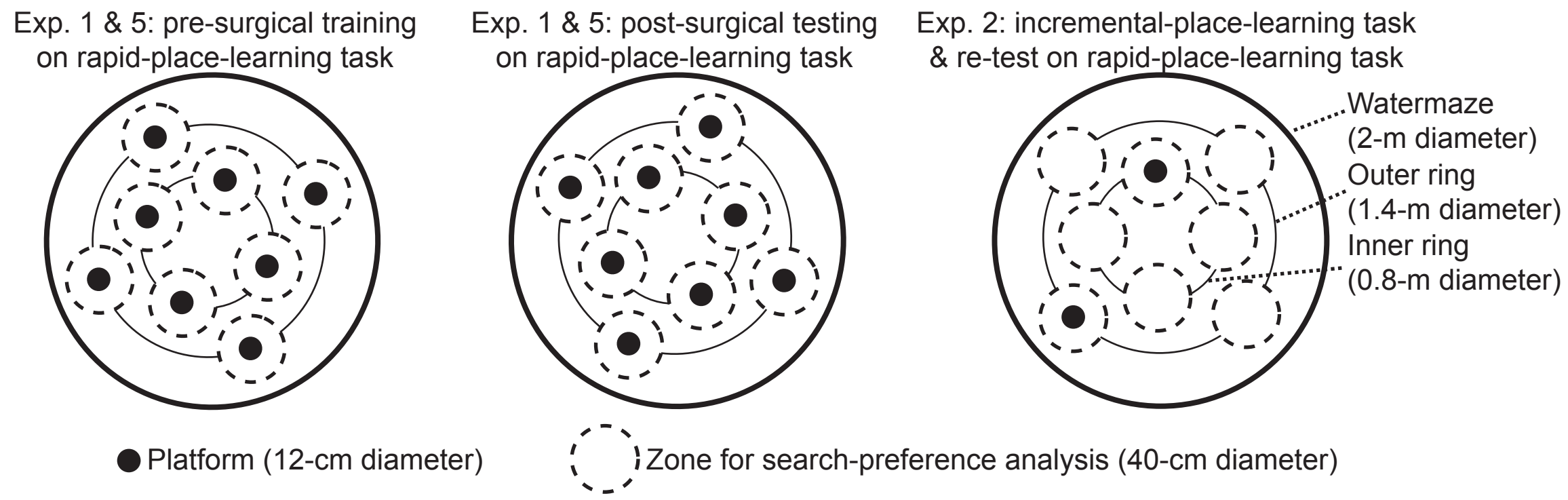
Exp. 1 & 5: pre-surgical training on rapid-place-learning task
Exp. 1 & 5: post-surgical testing on rapid-place-learning task
Exp. 2: incremental-place-learning task & re-test on rapid-place-learning task
Watermaze (2-m diameter)
Outer ring (1.4-m diameter)
Inner ring (0.8-m diameter)
Platform (12-cm diameter)
Zone for search-preference analysis (40-cm diameter)
