# Supplementary material for: From Rapid Place Learning to Behavioral Performance: A Key Role for the Intermediate Hippocampus
Source: PLoS Biol. 2009 Apr 21;7(4):e1000089. doi: 10.1371/journal.pbio.1000089 (PMC2671558; doi:10.1371/journal.pbio.1000089)
Supplement: Supplementary file 10 [file pbio.1000089.sd001.doc]

**SUPPLEMENTARY TEXT (Bast et al., The intermediate hippocampus . . .)**

- Supplementary Results 1-2 (p. 2-4)

- Supplementary Discussion 1-2 (p. 5)

- Supplementary Materials and Methods (p. 6-9)

- Supplementary References (p. 10)

**Supplementary Results**

**1. Lesion analysis**

***Experiment 1: partial hippocampal lesions sparing intermediate, temporal, or septal hippocampus, and complete hippocampal lesions***

Brains with intended septal sparing, often showed some sizeable temporal sparing (2.88±1.6%; 0.0 to 6.3%); if this was larger than 10% of total hippocampal volume, the rat was excluded from all analysis (8 out of 21 rats; another rat intended for the septal sparing group died during surgery). Rats with intended sparing centering on the intermediate hippocampus were excluded, if the septal tip (ca. 1.8 and 3.3 mm posterior from bregma in [1,2]) or the temporal tip (ca. 4.2 to 5.8 posterior from bregma) did not show nearly complete damage of all subfields (2 out of 16 rats). Subicular sparing was not calculated as part of the hippocampal sparing to allow comparison with previous studies [3-7], but was estimated separately based on measurements in four rats per group (with hippocampal sparing close to the group mean). This was done as described for the hippocampal volume (**Materials and Methods**), except that a higher magnification (20X) was used in the subiculum where discrimination of lesioned from intact tissue is more difficult than in the hippocampus due to the more diffuse cell distribution. In the group with temporal sparing, there was only minimal damage to the subiculum, the volume of which was ca. 93% of that in the sham group. The groups with intermediate and septal sparings showed comparable subicular damage, with only ca. 54% or 49%, respectively, spared. Lesions of the complete hippocampus left only ca. 10% of the subiculum intact. It is important to note that group differences in subicular sparing cannot account for the performance differences found in the present study (**Fig. 2, Fig. 3B,C**). Thus, rats with hippocampal tissue restricted to an intact intermediate hippocampus showed the best performance, while rats with hippocampal residuals at the septal pole were markedly impaired, even though both groups had comparable subicular damage of about 50%. Moreover, rats with hippocampal residuals at the temporal pole showed marked performance deficits even though they had only minimal damage (<10%) to the subiculum. Finally, there was occasional minor damage to the neocortex overlying the hippocampus, and - in those groups where some of the ibotenate injections targeted the temporal tip of the hippocampus - to the entorhinal cortex beneath the hippocampus.

***Experiment 5: lesions to the intermediate hippocampus***

One out of the 12 rats with an intended lesion to the intermediate hippocampus was excluded, as there was a small tissue bridge in the left hippocampus linking the temporal and septal tip. Subicular sparing in rats with lesions to the intermediate hippocampus was approximately 68% of that in the sham group (estimated based on measurements in four rats per group, which had hippocampal sparing close to the group mean). The subicular damage cannot account for the poor performance of rats with lesions to the intermediate hippocampus (**Fig. 6C**), as in Experiment 1 and 2 the group with a hippocampal residual in the intermediate region showed good performance in spite of only about 54% of subicular sparing (**Fig. 2, Fig. 3B,C**). Rats with lesions to the intermediate hippocampus typically sustained some damage to neocortical areas (most posterior part of parietal cortex, most anterior part of the visual cortex) overlying the posterior parts of the hippocampus [ca. 4.2-6.8 mm posterior from bregma according to [1,2]].

**2. Swim speed**

Analysis focused on the first trial in the watermaze following surgery, when differences in behavior due to memory for certain locations were minimal (as the previous location, from the last day of pre-surgical training, has basically been forgotten, and a new location not yet been learnt). In experiment 1, swim speed in the groups with lesions to the temporal tip of the hippoampus [i.e., with the intermediate region spared (27.62±0.91 cm/s), only the septal pole intact (26.15±1.20 cm/s), and with complete hippocampal lesions (28.75±0.76 cm/s)] was significantly higher (p<0.005) as compared to sham-operated rats (mean±SEM: 22.50±0.74 cm/s) and the groups with the temporal pole of the hippocampus spared (22.670±0.73 cm/s) (main effect of groups: F_4,84_=11.41, p<0.0001; no further significant differences between groups based on post-hoc comparisons: p>0.06). Importantly, the group differences in swim-speed are unrelated to the differences in task performance observed in the present study (**Fig. 2D,E, Fig. 3B,C**). In experiment 6, rats with lesions to the intermediate hippocampus, damaging the temporal hippocampus except for the 20% at the tip, tended to swim faster (25.32±0.85 cm/s) than the sham-operated group (23.09±0.72 cm/s) (t_21_=2.02, p=0.057).

**Supplementary Discussion**

**1. Delay-independent impairments on one-trial place learning task following hippocampal lesions**

Our findings confirm a previous study showing rats with complete hippocampal lesions to be impaired on the delayed-matching-to-place task in the watermaze with a retention delay of 15 s and 20 min [8]. Furthermore, delay-independent deficits in rats with hippocampal lesions have been found in dry-land delayed-matching-to-place tasks [9,10]. Recent studies in humans with hippocampal damage also revealed marked impairments on tests requiring rapid learning of stimuli and their relations, including place information, even at very short retention delays of a few seconds [11-14].

**2. Rapid vs. incremental learning**

Rats with complete hippocampal lesions have also been reported to show intact performance on a radial arm-maze task, if only incremental place learning was necessary, but not if rapid place learning was required [15], and to acquire normal freezing to a context across multiple context-shock pairings, but not within one trial [16].

**Supplementary Materials and Methods**

**Single-unit recording experiments**

Upon recovery from the surgery, rats were introduced for the first time to the recording environment and the 1 m X 1 m arena. The implanted microdrives were connected via a lightweight cable to a series of electronic components (Axona Ltd, UK) passing the single-unit information to a computer for storage and analysis. The neuronal signal was first passed through an RC-coupled, unity-gain operational amplifier located at the base of the cable nearest to the rat’s head. The signal from each channel was then amplified 5,000 to 13,000 times and bandpass filtered (360-6000Hz) and recorded differentially against a channel on a separate tetrode. The rats were screened daily for cells as they learned to randomly explore the familiar environment (Fam) in search of wet rice Krispies, scattered to the arena floor by the experimenter from behind a black curtain. The rat’s position was tracked with a camera mounted above the arena and light-emitting diodes located on the cable next to the rat’s head. Position samples of these lights were taken at 50 Hz.

Experimental recordings began in the familiar environment once putative complex-spike cells were encountered (a formal isolation of single-units was conducted off-line, see below). One channel without clear single-unit signals was dedicated to EEG recordings during experiments (band-pass filtered below 500 Hz). In recording the activity of single-units, each channel was monitored every 20 μs, and a channel whose electric potential reached a pre-determined threshold (55-65 μV) triggered the recording of 50 samples during 1 ms at 32 kHz for all channels of that tetrode. These digitized spike waveforms were stored on the computer, alongside position-tracking data and the time since the start of the recording.

Recording trials in the familiar environment included typically ca. 15 min of random search for rice Krispies. Between trials, the rat was placed in a holding bucket for typically 30 min to 1 h of rest. The cable was untwisted if necessary at this time, and the arena floor was cleaned with a mixture of soap, alcohol, and water to remove cryptic olfactory cues. The rat was replaced into the center of the familiar arena for a second recording trial. This process was repeated several times to test stability of the neurons and spatial representations. On subsequent days, when new cells were evident, recordings in the familiar environment were repeated.

Recording sequences involving the new environment (New) began after at least three simultaneously-recorded neurons exhibited stable spatial representations in the familiar environment. The recording sequence of Fam-New-New-Fam consisted of ca. 15-min recording trials interleaved with approximately 10-min pauses in the holding bucket for cleaning and changing of the environment.

**Off-line single-unit isolation and analysis**

Cells were analyzed with custom-made scripts using Matlab software (Steven Huang, University of Edinburgh, UK; The Mathworks, Inc., UK) in conjunction with KlustaKwik [17] and Klusters [18] ([http://klusters.sourceforge.net](http://klusters.sourceforge.net/)) for sorting spikes into individual cells. Spikes were classified using features of their waveforms: energy, first principal component, peak, width, and peak time. Well-isolated pyramidal cells, identified by auto-correlations indicative of bursting activity (a shoulder surrounding the refractory period of at least 3 times the mean firing rate), a refractory period of ±2 ms, and a negative after-hyperpolarization longer than 300 µs were taken for further analyses. To eliminate spikes occurring during sharp waves, analysis was restricted to spikes occurring while the rat was moving faster than 2 cm/s. Furthermore, only neurons with an average firing rate greater than 0.2 Hz on at least one trial were analyzed in detail.

Using the recorded spike data and position information, several measures were calculated for each neuron: average firing rate across the entire trial; the peak firing rate (highest mean firing rate of all 5 cm X 5 cm pixels contained within the recording environment), as well as the spatial information content in bits per spike and the sparsity of firing over the arena [19].

Firing-rate maps for each trial were produced from the rat’s path and the neuron’s spike data. The recording areas in the familiar arena (1 m X 1 m) and the new arena (0.64 m X 0.94 m) were divided into 5 cm X 5 cm pixels, and the firing rate for each pixel was calculated as the number of spikes per time the rat spent in that pixel. Note that the novel environment was bounded by a small rim, not a tall wall, so that the area available for exploration by the rat, and hence the recording area, was slightly larger than the arena itself. Only pixels visited by the rat for a minimum of 20 ms were assigned a firing rate. For preparation of the figures, firing-rate maps were smoothed by a shading-interpolation function in Matlab (The Mathworks, Inc., UK).

To compare firing patterns on different trials, Pearson’s correlations between the firing-rate maps were calculated. To calculate correlations between firing patterns on trials in the familiar and the new environment, the number of pixels in the rate maps of the two environments had to be equalized. This was done by two different methods. The first focused on spatial location with respect to the room, in which the arenas were placed, and involved trimming the firing-rate map from the familiar environment slightly in the height dimension. The second method focused on the patterns arising from the shape of the arenas and involved enlarging the map from the new arena slightly in the height dimension, so that each map was divided into ca. 20 X 20 pixels. Both methods of analysis yielded similar correlation results, and analysis based on room location was chosen for presentation in the paper.

Lastly, to examine firing-rate stability and positional stability of place fields in the familiar environments, the change between trials in both average firing rates and in peak firing rates [20] was calculated as: absolute value of [(firing rate1 – firing rate 2) / (firing rate1 + firing rate 2)], and the movement of firing peak (i.e., change in pixel location of the peak firing rate) was calculated by: SQRT [(change in X-coordinate)^2^ + (change in Y-coordinate)^2^].

**Supplementary References**

1. Paxinos G, Watson, C. (1998) The rat brain in stereotaxic coordinates. San Diego, CA: Academic Press.

2. Swanson LW (2004) Brain maps: structure of the rat brain. San Diego, CA: Academic Press.

3. Moser MB, Moser EI, Forrest E, Andersen P, Morris RG (1995) Spatial learning with a minislab in the dorsal hippocampus. Proc Natl Acad Sci U S A 92: 9697-9701.

4. Kjelstrup KG, Tuvnes FA, Steffenach HA, Murison R, Moser EI, et al. (2002) Reduced fear expression after lesions of the ventral hippocampus. Proc Natl Acad Sci U S A 99: 10825-10830.

5. de Hoz L, Knox J, Morris RG (2003) Longitudinal axis of the hippocampus: both septal and temporal poles of the hippocampus support water maze spatial learning depending on the training protocol. Hippocampus 13: 587-603.

6. Broadbent NJ, Squire LR, Clark RE (2004) Spatial memory, recognition memory, and the hippocampus. Proc Natl Acad Sci U S A 101: 14515-14520.

7. Bannerman DM, Deacon RM, Offen S, Friswell J, Grubb M, et al. (2002) Double dissociation of function within the hippocampus: spatial memory and hyponeophagia. Behav Neurosci 116: 884-901.

8. Steele RJ, Morris RG (1999) Delay-dependent impairment of a matching-to-place task with chronic and intrahippocampal infusion of the NMDA-antagonist D-AP5. Hippocampus 9: 118-136.

9. Kesner RP (1994) Hippocampus and memory for time. Behav Brain Sci 17: 485-486.

10. Gilbert PE, Kesner RP, DeCoteau WE (1998) Memory for spatial location: role of the hippocampus in mediating spatial pattern separation. J Neurosci 18: 804-810.

11. Hannula DE, Tranel D, Cohen NJ (2006) The long and the short of it: relational memory impairments in amnesia, even at short lags. J Neurosci 26: 8352-8359.

12. Olson IR, Page K, Moore KS, Chatterjee A, Verfaellie M (2006) Working memory for conjunctions relies on the medial temporal lobe. J Neurosci 26: 4596-4601.

13. Ranganath C, Blumenfeld RS (2005) Doubts about double dissociations between short- and long-term memory. Trends Cogn Sci 9: 374-380.

14. Hartley T, Bird CM, Chan D, Cipolotti L, Husain M, et al. (2007) The hippocampus is required for short-term topographical memory in humans. Hippocampus 17: 34-48.

15. Hunt ME, Kesner, R.P., Evans, R.B. (1994) Memory for spatial location: functional dissociation of entorhinal cortex and hippocampus. Psychobiology 22: 186-194.

16. Wiltgen BJ, Sanders MJ, Anagnostaras SG, Sage JR, Fanselow MS (2006) Context fear learning in the absence of the hippocampus. J Neurosci 26: 5484-5491.

17. Harris KD, Henze DA, Csicsvari J, Hirase H, Buzsaki G (2000) Accuracy of tetrode spike separation as determined by simultaneous intracellular and extracellular measurements. J Neurophysiol 84: 401-414.

18. Hazan L, Zugaro M, Buzsaki G (2006) Klusters, NeuroScope, NDManager: a free software suite for neurophysiological data processing and visualization. J Neurosci Methods 155: 207-216.

19. Markus EJ, Barnes CA, McNaughton BL, Gladden VL, Skaggs WE (1994) Spatial information content and reliability of hippocampal CA1 neurons: effects of visual input. Hippocampus 4: 410-421.

20. Wilson IA, Ikonen S, Gallagher M, Eichenbaum H, Tanila H (2005) Age-associated alterations of hippocampal place cells are subregion specific. J Neurosci 25: 6877-6886.
