# Supplementary material for: From Rapid Place Learning to Behavioral Performance: A Key Role for the Intermediate Hippocampus
Source: PLoS Biol. 2009 Apr 21;7(4):e1000089. doi: 10.1371/journal.pbio.1000089 (PMC2671558; doi:10.1371/journal.pbio.1000089)
Supplement: Supplementary file 11 [file pbio.1000089.st001.doc]

**Table S1:** Firing properties of complex spike cells in septal CA1 recorded from control rats with an intact hippocampus and from rats with partial hippocampal lesions sparing only the septal pole.

| **Group/Rat/**  **[nr. of cells]** | **Average firing rate (Hz)** | **Peak firing rate**  **(Hz)** | **Spatial info (bits/spk)** | **Sparsity** | **Stability Fam-Fam** | | | |
| --- | --- | --- | --- | --- | --- | --- | --- | --- |
|  |  |  |  |  | *Correl.* | *Firing-rate changes* | | *Movement of firing peak (cm)* |
|  |  |  |  |  |  | *Aver. rate* | *Peak rate* |  |
|  |  |  |  |  |  |  |  |  |
| ***Control*** [28] |  |  |  |  |  |  |  |  |
| #4 [9] | 0.67 | 10.07 | 1.82 | .21 | .88 | .16 | .18 | 24.0 |
| #11 [1] | 0.84 | 6.05 | 1.13 | .30 | .93 | .14 | .05 | 5.0 |
| #12 [5] | 4.29 | 20.89 | .90 | .42 | .84 | .25 | .17 | 15.5 |
| #14 [13] | 1.25 | 11.60 | 1.70 | .27 | .81 | .13 | .14 | 21.5 |
| **Mean±SEM** | **1.79±0.85** | **12.39±3.13** | **1.39±0.22** | **.30±.05** | **.88±.02** | **.17±.03** | **.14±.03** | **16.5±4.2** |
|  |  |  |  |  |  |  |  |  |
|  |  |  |  |  |  |  |  |  |
| ***Lesioned*** [42] |  |  |  |  |  |  |  |  |
| #3 [2] | 1.68 | 13.46 | 1.61 | .26 | .74 | .62 | .33 | 16.3 |
| #5 [6] | 1.35 | 15.70 | 2.06 | .23 | .80 | .27 | .31 | 7.4 |
| #10 [34] | 1.64 | 9.79 | 0.97 | .43 | .71 | .24 | .21 | 19.2 |
| **Mean±SEM** | **1.56±0.10** | **12.98±1.72** | **1.55±0.32** | **.31±.06** | **.75±.03*** | **.38±.12** | **.28±.04*** | **14.3±3.6** |
|  |  |  |  |  |  |  |  |  |

The values for individual rats represent the mean of all cells recorded in this rat during trials in the familiar arena. Firing rates, spatial information, and sparsity were calculated from the first trial in the familiar arena during which the individual cell was recorded. The stability measures were calculated from firing rates of the first and the second recording trials of individual cells in the familiar arena. The firing-rate and spike-path maps for the recording trials based on which the table is calculated are depicted in **Supplementary Fig. 5A** and **B**.

*Significantly different from Control: t_5_>3.0, p<0.03; all other comparisons Control vs. Lesioned: t_5_<2.0, p>0.11.

**Supplementary Table 2:** Stereotaxic coordinates (in mm from bregma) and injection volumes for the hippocampal ibotenic-acid injections in the different lesion groups.

| **Group / coordinates** | | | Injected volume (µl) |
| --- | --- | --- | --- |
| AP | ML | DV |  |
| *40% intermediate sparing* | | |  |
| -2.4  -3.0  -4.3  -4.8  -5.7 | ±1.0  ±1.4  ±3.0  ±4.0  ±3.9  ±3.9 | 3.0  2.7  2.1, 2.9  7.0  7.0  7.0 | 0.05  0.10  0.05, 0.05  0.10  0.10  0.05 |
|  |  |  |  |
| *40% temporal sparing* | | |  |
| -2.4  -3.0  -4.0  -5.0 | ±1.0  ±1.4  ±3.0  ±3.7  ±2.6  ±5.0  ±3.9 | 3.0  2.7  2.1, 2.9  2.7  1.8, 2.8  4.0  3.1 | 0.05  0.10  0.05, 0.05  0.10  0.05, 0.05  0.05  0.05 |
|  |  |  |  |
| *40% septal sparing* | | |  |
| -4.8  -5.0  -5.7  -6.15 | ±3.9  ±5.4  ±3.0  ±3.9  ±5.0  ±4.6 | 7.0  5.1  3.0  3.6, 7.0  3.6, 4.9  4.6 | 0.10  0.10  0.05  0.05, 0.05  0.05, 0.05  0.05 |
|  |  |  |  |
| *Complete hippocampal lesion* | | |  |
| -2.4  -3.0  -4.0  -4.3  -4.9  -5.9 | ±1.0  ±1.4  ±3.0  ±2.6  ±3.7  ±4.0  ±3.9  ±4.3  ±5.1 | 3.0  2.1, 2.9  2.7  1.8, 2.8  2.7  7.0  3.5, 7.0  3.9  4.5  5.3 | 0.05  0.05, 0.05  0.10  0.05, 0.05  0.10  0.05  0.05, 0.10  0.10  0.08  0.08 |
|  |  |  |  |
| *Lesion to intermediate hippocampus (sparing 20% each at septal and temporal tip)* | | |  |
| -4.5  -5.0  -5.7    -6.15 | ±2.3  ±3.6  ±3.9  ±5.0  ±3.9  ±5.0  ±4.6 | 1.9, 2.8  2.7  3.1  4.0  3.6  3.6, 4.9  4.6 | 0.05, 0.05  0.05  0.05  0.05  0.05  0.05, 0.05  0.10 |

**Supplementary Figures:**

**Supplementary Fig. 1:** **Experiment 1: pre-training performance in the five prospective surgical groups at the two different retention delays between trial 1 and 2.**

The prospective surgical groups, to receive different hippocampal lesions or sham surgery (see **Fig. 1c**), were matched for: **(A)** % time in correct zone (mean±SEM; chance level 12.5%, indicated by stippled line), when trial 2 was run as a probe on days 4 and 8; **(B)** latencies (mean±SEM) to reach the platform, averaged across days (all relevant main effects: F<1; interactions: F<1.5, p>0.14). At this stage of the experiment, there was no significant dependence of performance measures on the delays between trial 1 and 2 (all relevant main effects: F<1.1, p>0.30; interactions: F<2.6, p>0.05). In all groups, performance based on one-trial place memory was indicated by a %time in correct zone about twice as high as expected based on chance and a significant latency reduction from trial 1 to 2 (all t>5.3, p<0.0005).

**Supplementary Fig. 2:** **Experiment 1: post-surgical performance on the rapid-place learning task with a 15- to 30-s or a 20-min delay between trial 1 and 2.**

**(A)** % time in the correct zone (mean±SEM; chance level 12.5%, indicated by stippled line) when trial 2 was conducted as a probe trial either 15-30 s or 20 min after trial 1 (one probe per delay). Apart from the significant main effect of group (see main text), there was a significant main effect of delay (F_1,84_=6.10, p<0.02), independent of group (interaction: F_4,84_=1.45, p>0.2), indicating forgetting of 1-trial place memory.

**(B)** Path lengths (mean±SEM) to reach the platform, shown separately for the days with a trial1-trial 2 delay of 10-30 s and 20 min (four days at each delay). Apart from the trial-dependent group differences (see main text), there was a main effect of delay (F_1,84_=8.32, p<0.005), but only a strong tendency for this effect to be trial-dependent (F_3,252_=2.44, p=0.06); the effects and interactions involving delay were group independent (F<1.63, p>0.17). That % time in correct zone during pre-surgical testing (Supplementary Fig. 1) did not show delay-dependence may reflect that one of the probe trials was run on day 4, when performance had not quite yet reached asymptotic levels.

**Supplementary Fig. 3:** **Experiment 2: performance on the incremental-place-learning task across all days and trials.**

**(A)** Path lengths (mean±SEM) to reach the platform on the eight daily trials (T1-8) of days 1-5 and on the single trial on day 6.

**(B)** % time in correct zone (mean±SEM; chance level 12.5%, indicated by stippled line) on the trials which were run as probes (T2 and 6 from days 1 to 5 and T1 on day 6).

The group differences in both measures decreased across trials within days and, especially, between days. This was supported by highly significant group x trial interactions (path lengths: F_160,3360_=1.69, p<0.0001; search preference: F_40,840_=2.28, p<0.0001).

**Supplementary Fig. 4:** **Experiment 2: path lengths and savings during re-testing on the rapid- place-learning task.**

Only sham-operated rats and the group with hippocampal lesions sparing the intermediate region showed a significant reduction in path lengths from trial 1 to 2 (t>3.54, p<0.001; all other t<1.23, p>0.24), i.e. ‘savings’ (group x trial interaction for path lengths: F_12,201_= 3.91, p<0.0001; main effect of group on savings: F_4,67_=6.28, p<0.0003). Group differences: * different from sham-operated group and group with only the intermediate hippocampus spared (p<0.015); # different from group with only the intermediate hippocampus spared (p<0.03).

**Supplementary Fig. 5:** **Experiment 4: firing-rate and path maps for all cells recorded from septal CA1 during two successive recording trials in the familiar environment (Fam)***.*

Recordings from rats with hippocampal residuals at the septal pole **(A)** and from rats with an intact hippocampus **(B)** are shown. The color-coded firing-rate maps and, below, the location of spikes (red) on the rat’s trajectory (gray) are shown. The peak firing rate for each trial is indicated in red numbers above and the correlation between the firing-rate maps for the two successive trials is shown in black below. Quantitative measures of the firing properties of all cells in both groups of rats are summarized in **Supplementary Table 1**.

**Supplementary Fig. 6:** **Experiment 4: firing-rate and path maps for all cells recorded from septal CA1 during a succession of 15-min trials in the familiar environment (Fam) and the new environment (New).**

Recordings from hippocampal residuals at the septal pole, left, and from an intact hippocampus, right, are shown. The color-coded firing-rate maps and, below, the location of spikes (red) on the rat’s trajectory (gray) are shown. All firing rate maps are scaled to the same maximum firing rate, equal to the maximum of all four trials and indicated in red to the right; correlations between the firing rate maps for different trials are indicated in black below.

**Supplementary Fig. 7:** **Experiment 5: pre-surgical training on the rapid-place-learning task and performance matching.**

**(A)** Latencies (mean±SEM) to reach the platform on the four daily trials across the eight days of pre-surgical training (data are shown for the 23 rats that were included in the final analysis). Data were collapsed for the two different delays between trial 1 and 2 (15-30 s or 20 min), which were used equally often and for half of the rats each on every day. On days 4 and 8, trial 2 was run as probe.

(**B** and **C**) The prospective surgical groups, to receive sham surgery (n=12) or lesions to the intermediate hippocampus (n=11), were matched for: **(B)** % time in correct zone (mean±SEM; chance level 12.5%, indicated by stippled line), when trial 2 was run as a probe on days 4 and 8; **(C)** latencies (mean±SEM) to reach the platform, averaged across days (all relevant main effects: F < 1; interactions: F < 2.2, p>0.15). There was no significant dependence of performance measures on the delays between trial 1 and 2 (all relevant main effects: F < 1; interactions: F < 2.0, p>0.17). In both groups, performance based on one-trial place memory was indicated by a significant latency reduction from trial 1 to 2, and a %time in correct zone about twice as high as expected based on chance (t_11_ > 4.28, p<0.002).

**Supplementary Fig. 8:** **Experiment 5: post-surgical performance on the rapid-place learning task with a 15- to 30-s or a 20-min delay between trial 1 and 2.**

**(A)** % time in the correct zone (mean±SEM; chance level 12.5%, indicated by stippled line) when trial 2 was conducted as a probe trial either 15-30 s or 20 min after trial 1 (one probe per delay). There was only a significant main effect of group (see main text), but no main effect of delay nor an interaction group X delay (both F_1,22_<1).

**(B)** Path lengths (mean±SEM) to reach the platform, shown separately for the days with a trial1-trial 2 delay of 10-30 s and 20 min (four days at each delay).

Apart from the trial-dependent group differences (see main text), there were no significant main effects or interactions (all F<2.3, p>0.09).

**Supplementary Fig. 9:** **Platform locations used during the different stages of behavioral testing and the surrounding zones used for the analysis of search preference.**

**Supplementary Movies 1-5:** Movies 1-5 show rotations of the 3D reconstructions depicted in **Fig. 1C and 6B**; please note, different from the figures, hippocampal residuals are not overlaid on an intact hippocampus.

**Supplementary Movie 1**: Intact hippocampus.

**Supplementary Movie 2:** Hippocampal residual in the intermediate region.

**Supplementary Movie 3:** Hippocampal residual at the temporal pole.

**Supplementary Movie 4:** Hippocampal residual at the septal pole.

**Supplementary Movie 5:** Hippocampal residuals at the temporal and septal tip following selective lesion of the intermediate region.
