# Supplementary material for: From Rapid Place Learning to Behavioral Performance: A Key Role for the Intermediate Hippocampus
Source: PLoS Biol. 2009 Apr 21;7(4):e1000089. doi: 10.1371/journal.pbio.1000089 (PMC2671558; doi:10.1371/journal.pbio.1000089)
Supplement: Supplementary file 12 [file pbio.1000089.st002.doc]

**Table S2:** Stereotaxic coordinates (in mm from bregma) and injection volumes for the hippocampal ibotenic-acid injections in the different lesion groups.

| **Group / coordinates** | | | Injected volume (µl) |
| --- | --- | --- | --- |
| AP | ML | DV |  |
| *40% intermediate sparing* | | |  |
| -2.4  -3.0  -4.3  -4.8  -5.7 | ±1.0  ±1.4  ±3.0  ±4.0  ±3.9  ±3.9 | 3.0  2.7  2.1, 2.9  7.0  7.0  7.0 | 0.05  0.10  0.05, 0.05  0.10  0.10  0.05 |
|  |  |  |  |
| *40% temporal sparing* | | |  |
| -2.4  -3.0  -4.0  -5.0 | ±1.0  ±1.4  ±3.0  ±3.7  ±2.6  ±5.0  ±3.9 | 3.0  2.7  2.1, 2.9  2.7  1.8, 2.8  4.0  3.1 | 0.05  0.10  0.05, 0.05  0.10  0.05, 0.05  0.05  0.05 |
|  |  |  |  |
| *40% septal sparing* | | |  |
| -4.8  -5.0  -5.7  -6.15 | ±3.9  ±5.4  ±3.0  ±3.9  ±5.0  ±4.6 | 7.0  5.1  3.0  3.6, 7.0  3.6, 4.9  4.6 | 0.10  0.10  0.05  0.05, 0.05  0.05, 0.05  0.05 |
|  |  |  |  |
| *Complete hippocampal lesion* | | |  |
| -2.4  -3.0  -4.0  -4.3  -4.9  -5.9 | ±1.0  ±1.4  ±3.0  ±2.6  ±3.7  ±4.0  ±3.9  ±4.3  ±5.1 | 3.0  2.1, 2.9  2.7  1.8, 2.8  2.7  7.0  3.5, 7.0  3.9  4.5  5.3 | 0.05  0.05, 0.05  0.10  0.05, 0.05  0.10  0.05  0.05, 0.10  0.10  0.08  0.08 |
|  |  |  |  |
| *Lesion to intermediate hippocampus (sparing 20% each at septal and temporal tip)* | | |  |
| -4.5  -5.0  -5.7    -6.15 | ±2.3  ±3.6  ±3.9  ±5.0  ±3.9  ±5.0  ±4.6 | 1.9, 2.8  2.7  3.1  4.0  3.6  3.6, 4.9  4.6 | 0.05, 0.05  0.05  0.05  0.05  0.05  0.05, 0.05  0.10 |
